# Longer aftershocks duration in extensional tectonic settings

**DOI:** 10.1038/s41598-017-14550-2

**Published:** 2017-11-27

**Authors:** E. Valerio, P. Tizzani, E. Carminati, C. Doglioni

**Affiliations:** 1 Department of Earth Sciences, Sapienza University of Rome, Rome, Italy; 2 National Research Council (CNR), Istituto per il Rilevamento Elettromagnetico dell’Ambiente (IREA), Napoli, Italy; 3 Istituto Nazionale di Geofisica e Vulcanologia (INGV), Rome, Italy

## Abstract

Aftershocks number decay through time, depending on several parameters peculiar to each seismogenic regions, including mainshock magnitude, crustal rheology, and stress changes along the fault. However, the exact role of these parameters in controlling the duration of the aftershock sequence is still unknown. Here, using two methodologies, we show that the tectonic setting primarily controls the duration of aftershocks. On average and for a given mainshock magnitude (1) aftershock sequences are longer and (2) the number of earthquakes is greater in extensional tectonic settings than in contractional ones. We interpret this difference as related to the different type of energy dissipated during earthquakes. In detail, (1) a joint effect of gravitational forces and pure elastic stress release governs extensional earthquakes, whereas (2) pure elastic stress release controls contractional earthquakes. Accordingly, normal faults operate in favour of gravity, preserving inertia for a longer period and seismicity lasts until gravitational equilibrium is reached. Vice versa, thrusts act against gravity, exhaust their inertia faster and the elastic energy dissipation is buffered by the gravitational force. Hence, for seismic sequences of comparable magnitude and rheological parameters, aftershocks last longer in extensional settings because gravity favours the collapse of the hangingwall volumes.

## Introduction

**Figure 1:**
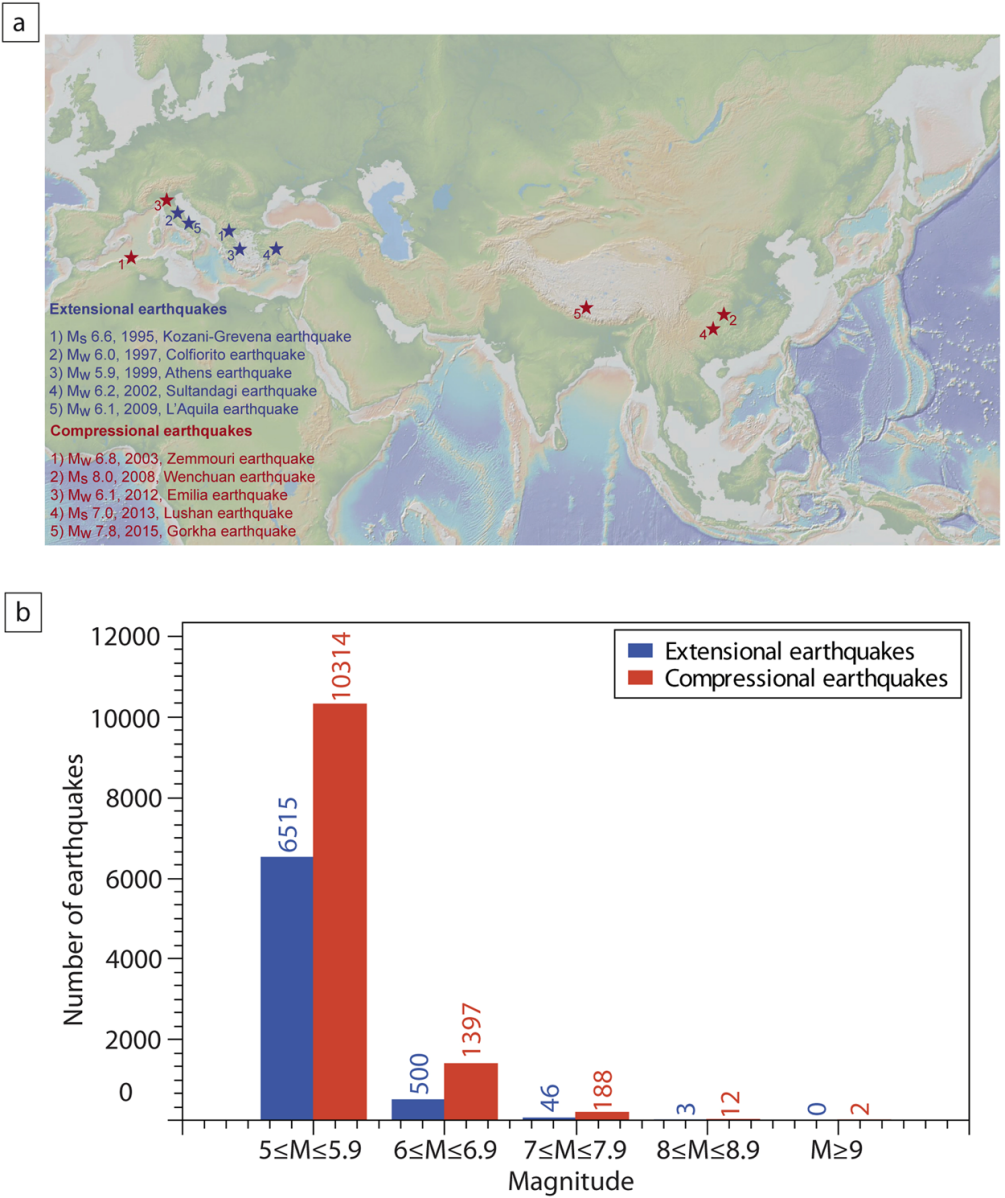
(**a**) Geographic location of the ten analysed case studies. Extensional earthquakes are shown in blue: 1) the M_s_ 6.6 Kozani-Grevena earthquake (1995, Greece), 2) the M_w_ 6.0 Colfiorito earthquake (1997, Central Italy), 3) the M_w_ 5.9 Athens earthquake (1999, Greece), 4) the M_w_ 6.2 Sultandagi earthquake (2002, Turkey), and 5) the M_w_ 6.3 L’Aquila earthquake (2009, Central Italy). Compressional earthquakes are shown in red: 1) the M_w_ 6.8 Zemmouri earthquake (2003, Algeria), 2) the M_s_ 8.0 Wenchuan earthquake (2008, China), 3) the M_w_ 6.1 Emilia earthquake (2012, Northern Italy), 4) the M_s_ 7.0 Lushan earthquake (2013, China), and 5) the M_w_ 7.8 Gorkha earthquake (2015, Nepal); the map was obtained by using the software GeoMapApp^63^ (e.g., www.geomapapp.com). (**b**) Histogram of the world earthquakes occurrence versus magnitude from 1976 to the present, in both extensional and compressional tectonic settings. Extensional compressional earthquakes are indicated in blue and red, respectively. We used the Global Harvard Centroid-Moment-Tensor (CMT) Catalog^6,7^ and selected seismic events (1976-to present) occurred down to 40 km depth and within the 5–10 magnitude range. To classify seismic events as thrust or normal events, we used the tension axis plunge and the null axis plunge values. This is due to the CMT Catalog report, which indicates that thrust faults have large plunge ( >45°) of tension axis, strike-slip faults have large plunge of null axis, and extensional faults have small plunge (<45°) for both tension and null axes.

Every day, moderate-to-large magnitude earthquakes release seismic energy stored within the Earth’s crust. This energy is accumulated for tens or thousands of years during the inter-seismic phase and released instantaneously (i.e., within seconds) through an earthquake (i.e., the mainshock) during the co-seismic phase^1–4^. After the mainshock, the energy release continues (for months to years) during the post-seismic phase in the form of aftershocks, generally characterized by magnitudes smaller than the mainshock^5^. A worldwide earthquake analysis using as data the Global Harvard Centroid-Moment-Tensor (CMT) Catalog^6,7^ shows that earthquakes within contractional tectonic settings are characterized by higher magnitude values (up to 9.5) than those within extensional tectonic settings (generally up to 7 with few exceptions; Fig. 1b). Schorlemmer *et al*.^8^ calculated the b-value of the Gutenberg-Richter law:

1 $$log\,N=a-bM$$

where *N* is the number of events within a certain range of magnitude, $a$ and $b$ are two costants and $M$ is the minimum magnitude. Their estimated b-value is equal to 1.1 for extensional and to 0.9 for contractional tectonic settings. Large earthquakes affect smaller crustal volumes in extensional tectonic settings than in contractional settings; this was proposed as the main reason for the occurrence of smaller magnitude earthquakes in rift zones^9^. On the contrary, few studies have focused on the control of the tectonic setting on the duration of aftershock sequences. A better understanding of the processes ruling the decay of aftershock with time (aftershocks decay) is fundamental to better constrain seismic hazard during ongoing seismic sequences.

Within a seismic sequence, seismological observations indicate that the aftershocks decay follows the Omori-Utsu law^10,11^:

2 $$n(t)=\frac{k}{{(c+t)}^{p}}$$

where *k* and *c* are constants, *t* is the time and *p* indicates the decay rate. The aftershocks decay also depends on several parameters peculiar to each seismogenic region, such as the tectonic setting (i.e., extensional, strike-slip, contractional regimes), the stress changes along fault, the structural heterogeneities, and the crustal rheology^12–14^. However, the geological and seismotectonic parameters that control the aftershocks decay during seismic sequences are still unclear^13^.

In this work, we focus on how the tectonic setting controls the aftershocks decay within seismic sequences. In particular, we analyse data from international catalogues with two different methods, comparing the aftershock sequences following five extensional settings mainshocks (Fig. 1a; Table 1) and five contractional settings mainshocks (Fig. 1a; Table 1). The average duration of aftershock sequences is longer, and the number of events is larger within extensional tectonic settings with respect to contractional tectonic settings. We propose an interpretation of these different behaviours in terms of differences in the orientation of forces acting during earthquake nucleation processes in the two settings.

**Table 1:** Case studies.

Earthquake	Occurrence	Magnitude	M_0_ (N * m)	Aftershocks sequence duration			Number of aftershocks	
				Tangents method	Mandelbrot method		Tangents method	Mandelbrot method
**Extensional earthquakes**								
Kozani-Grevena earthquake	13^rd^ May 1995	M_s_ 6.6	7.6 * 10^18^ ^15^	~300 days		~350 days	989	998
Colfiorito earthquake	26^th^ September 1997	M_w_ 6.0	1.2 * 10^18^ ^16^	~330 days		~330 days	2119	2119
Athens earthquake	7^th^ September 1999	M_w_ 5.9	1.0 * 10^18^ ^17^	~300 days		~400 days	281	295
Sultandagi earthquake	3^rd^ February 2002	M_w_ 6.2	2.4 * 10^18^ ^18^	~500 days		~540 days	922	944
L’Aquila earthquake	6^th^ April 2009	M_w_ 6.3	3.9 * 10^18^ ^19^	~515 days		~560 days	915	925
**Contractional earthquakes**								
Zemmouri earthquake	21^st^ May 2003	M_w_ 6.8	2.89 * 10^19^ ^20^	~100 days		~165 days	400	423
Wenchuan earthquake	12^th^ May 2008	M_s_ 8.0	9.4 * 10^20^ ^21^	~230 days		~230 days	1859	1859
Emilia earthquake	20^th^ May 2012	M_w_ 6.1	1.81 * 10^18^ ^22^	~110 days		~120 days	782	784
Lushan earthquake	20^th^ April 2013	M_s_ 7.0	1.01 * 10^19^ ^23^	~35 days		~35 days	122	122
Gorkha earthquake	25^th^ April 2015	M_w_ 7.8	7.55 * 10^20^ ^24^	~130 days		~130 days	786	786

List of ten case studies divided according to the fault kinematics. In this table we also reported earthquake characteristics (fault kinematics, occurrence, magnitude, seismic moment M_0_), the results obtained for each seismic sequence by using two different methods (e.g., the Tangents method and the Mandelbrot method) and the number of aftershocks occurred with M ≥ 2.5.

## Materials and Methods

### Collected data

In order to achieve comparable and homogeneous seismic sequences, we adopted the following criteria during the selection of earthquake sequences:

1. presence of local and well-distributed seismometric stations to avoid the occurrence of spatial gaps within the seismic sequence;
2. existence of complete seismological catalogues to avoid the occurrence of temporal lacks within the seismic sequence;
3. maximum hypocentral depth of 40 km (i.e., crustal earthquakes), thus excluding deeper subduction-related earthquakes;
4. mainshocks of M > 5.5 to obtain representative aftershock sequences;

We selected the following seismic sequences within extensional tectonic settings: Italian Central Apennines (1997, Colfiorito earthquake, M_w_ 6.0 and 2009, L’Aquila earthquake, M_w_ 6.3); Greece (1995, Kozani-Grevena earthquake, M_s_ 6.6, and 1999, Athens earthquake, M_w_ 5.9); central-western Turkey (2002, Sultandagi earthquake, M_w_ 6.2). Within contractional tectonic settings, we analysed earthquakes occurred within the following fold-and-thrust belts: Algerian Tell (2003, Zemmouri earthquake, M_w_ 6.8); Italian Northern Apennines (2012, Emilia earthquake, M_w_ 6.1); Nepalese Himalaya (2015 Gorkha earthquake, M_w_ 7.8); Chinese Sichuan Province (2008, Wenchuan earthquake, M_s_ 8.0, and 2013, Lushan earthquake, M_s_ 7.0).

We used seismic data presented in following catalogues: the INGV Earthquake Centre (National Institute of Geophysics and Volcanology, Italy, http://iside.rm.ingv.it, to analyse the 1997 Colfiorito, 2009 L’Aquila and 2012 Emilia sequences); the CENC (China Earthquake Network Center, China, http://www.csi.ac.cn/sichuan/index080512001.htm, to analyse the 2008 Wenchuan sequence, 2013 Lushan sequence and 2015 Gorkha sequences); the KOERI (Kandilli Observatory and Earthquake Research Institute, Turkey, http://www.koeri.boun.edu.tr, to analyse the 2002 Sultandagi sequence) and the NOA (National Observatory of Athens, Greece, http://www.gein.noa.gr, to analyse the 1995 Kozani-Grevena and the 1999 Athens sequences). We compared these data with those from the ISC^25^ (International Seismological Centre) catalogue. Specifically, in case of the 2003 Zemmouri sequence (Algeria), we only used seismological data included in the ISC catalogue because, at the moment, a local Algerian seismological catalogue is unavailable.

### Methods to determine the duration of aftershocks

**Figure 2:**
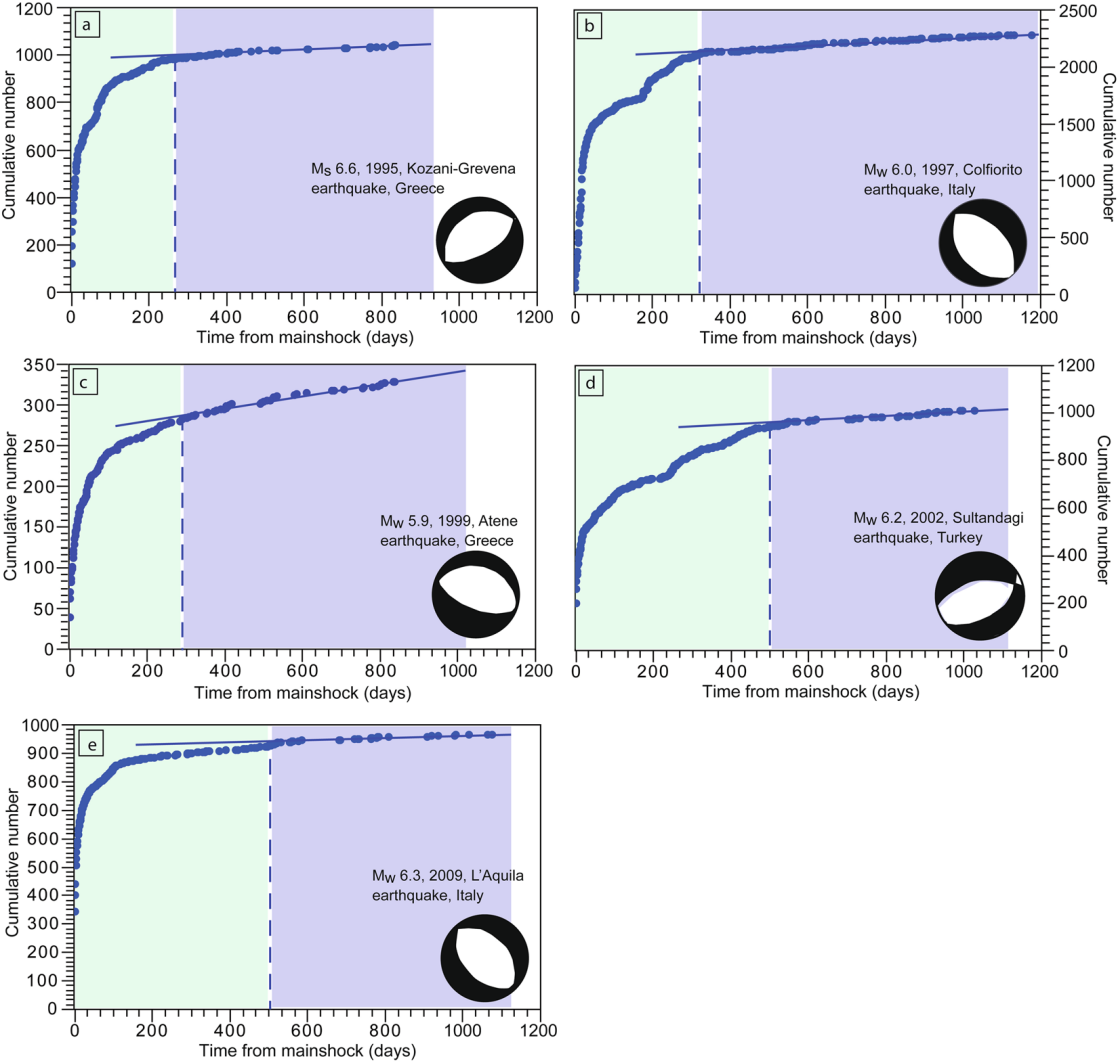
Aftershock sequences temporal evolution analysed by using the Tangents method, following five mainshocks nucleated in extensional areas: (**a**) the M_s_ 6.6 Kozani-Grevena earthquake (1995, Greece), (**b**) the M_w_ 6.0 Colfiorito earthquake (1997, Central Italy), (**c**) the M_w_ 5.9 Athens earthquake (1999, Greece), (**d**) the M_w_ 6.2 Sultandagi earthquake (2002, Turkey), and (**e**) the M_w_ 6.3 L’Aquila earthquake (2009, Central Italy). The cumulative number of earthquakes is shown versus the days from the mainshock nucleation. The blue dashed lines indicate the onsets of the linear trends, which are fitted by the blue solid lines. The light green and blue areas represent the seismicity related to the mainshock and the background seismicity, respectively.

**Figure 3:**
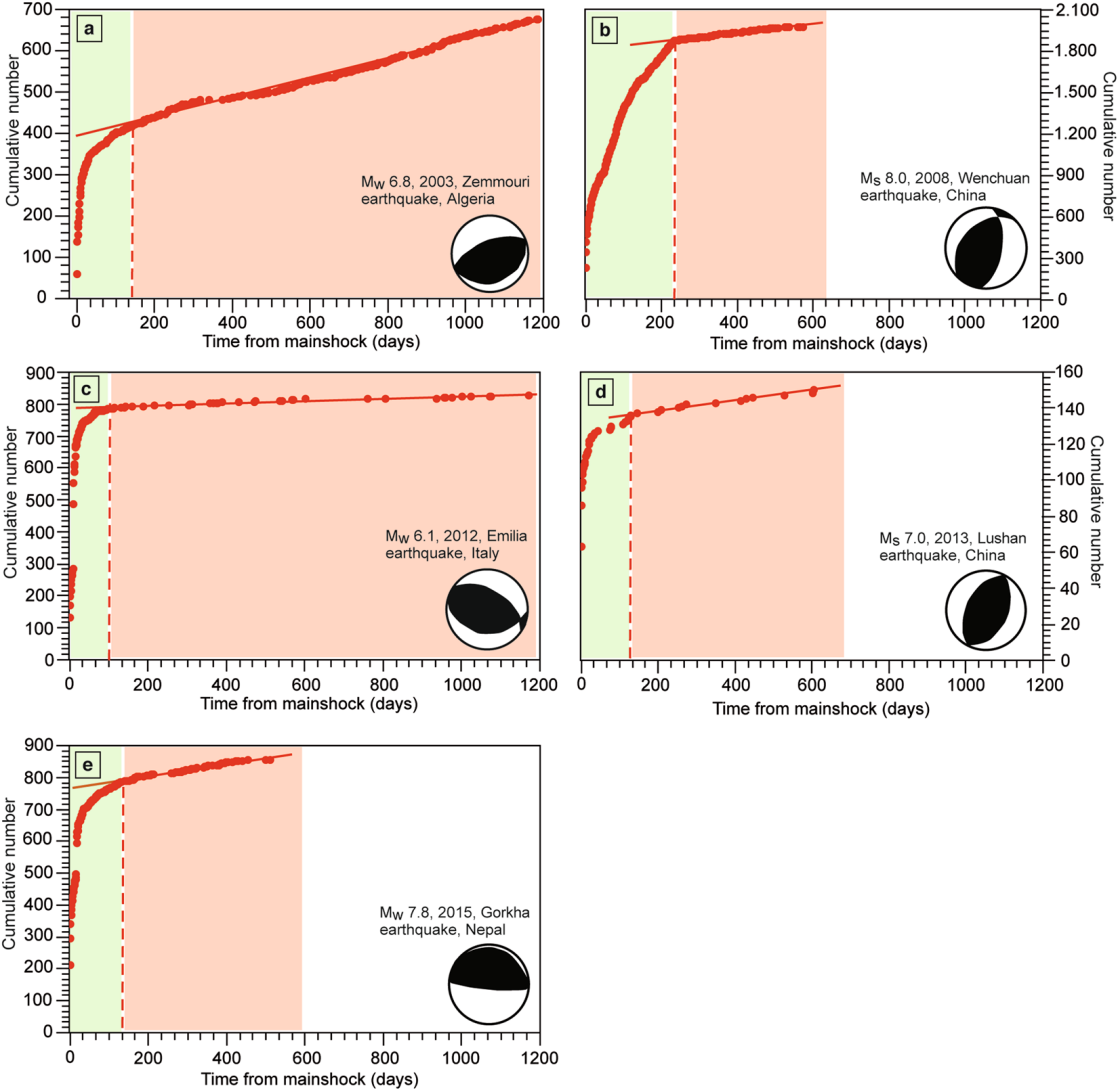
Aftershock sequences temporal evolution analysed by using the Tangents method, following five mainshocks nucleated in contractional areas: (**a**) the M_w_ 6.8 Zemmouri earthquake (2003, Algeria), (**b**) the M_s_ 8.0 Wenchuan earthquake (2008, China), (**c**) the M_w_ 6.1 Emilia earthquake (2012, Northern Italy), (**d**) the M_s_ 7.0 Lushan earthquake (2013, China), and (**e**) the M_w_ 7.8 Gorkha earthquake (2015, Nepal). The cumulative number of earthquakes is shown versus the days from the mainshock nucleation. The red dashed lines indicate the onsets of the linear trends, which are fitted by the red solid lines. The light green and red areas represent the seismicity related to the mainshock and the background seismicity, respectively.

In addition to the classical Omori law analysis performed to verify the completeness of the used seismic catalogues (results are reported in Figs S1 and S2 and Table S1 of the Supplementary Material), we employed two novel approaches to determine the duration and the number of events of the selected aftershock sequences:

1) Tangents method. We used the QtiPlot software to elaborate a statistical-descriptive analysis of seismic sequences. We adopted a completeness (threshold) magnitude (M_c_) of 2.5 as, below this threshold, the number of recorded seismic events strongly depends on the sensitivity of the seismic network, which varies from place to place. The adoption of a completeness magnitude, chosen on the basis of the minimum magnitude reported in each catalogue, allowed us to remove the seismic noise. The methodology is based on the comparison of the curves representing the cumulative number of earthquakes for each seismic sequence versus the days elapsed from the mainshock (Fig. 2). In principle, the lower the completeness magnitude, the greater the completeness of the curve. The corresponding curves present an initial non-linear increase followed by a linear one (Figs 2 and 3). The initial non-linear trend suggests that the seismic sequence related to the mainshock is still active, whereas the linear increment represents the characteristic background seismicity of the region. We consider the day when the tangent to the linear increment starts from the cumulative curve as indicative of the end of the aftershock sequence.

**Figure 4:**
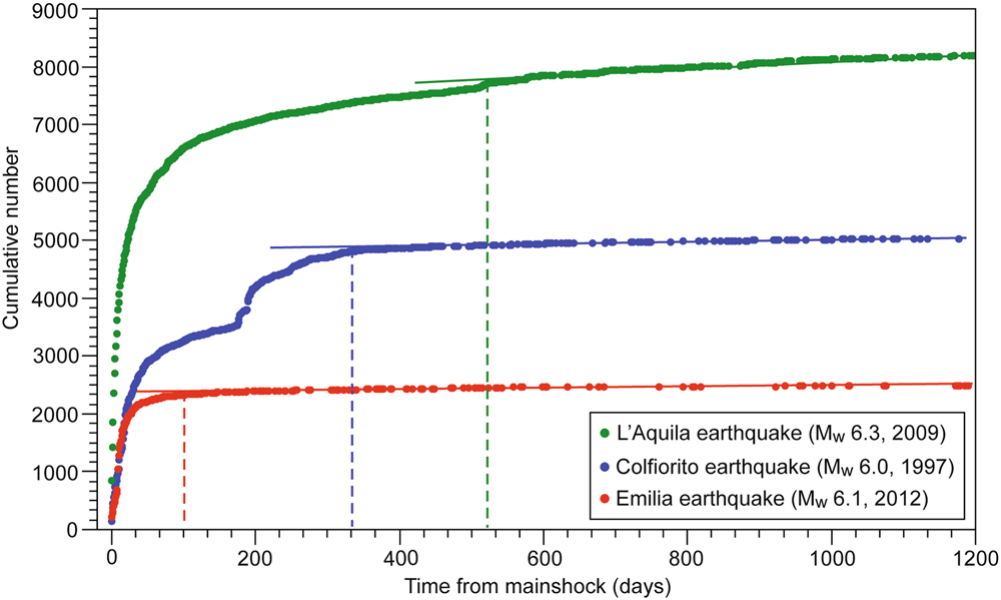
Comparison of aftershock sequences temporal evolution analysed by using the here proposed Tangents method in case of two Italian extensional earthquakes (the M_w_ 6.0 Colfiorito earthquake, 1997, Central Italy and the M_w_ 6.3 L’Aquila earthquake, 2009, Central Italy) and an Italian compressional earthquake (the M_w_ 6.1 Emilia earthquake, 2012, Northern Italy). The cumulative number of earthquakes is shown versus the days from the mainshock nucleation. The dashed lines indicate the onsets of the linear trends, which are fitted by the solid lines. The M_c_ is set at 1.6.

We used this analysis to compare the three selected Italian earthquakes: the 1997 (M_w_ 6.0) Colfiorito and the 2009 (M_w_ 6.3) L’Aquila extensional earthquakes and the 2012 (M_w_ 6.1) Emilia contractional earthquake, characterized by similar magnitudes and recorded by the same seismometric network (Fig. 4). In this case, we adopted a completeness magnitude (M_c_) of 1.6.

To understand the physical reason of the change of slope in the cumulative curves, we calculated the strain energy release ${{E}}_{s}$ generated by the earthquakes nucleation, which is related to the magnitude values *M* by the following empirical relation^26,27^ (Figs S3 and S4):

3 $$log\,{E}_{s}=1.44M+5.24$$

where ${{E}}_{s}$ is in Joule. In particular, we want to investigate the relationship between the energy released during the seismic sequence and the number of seismic events, represented by the cumulative curves.

2) Mandelbrot method. We used a numerical computing software to examine faulting and fragmentation processes using the fractals theory^28,29^. In this context, the fractal geometries are strictly related to the fragmentation processes caused by earthquake nucleation. The variation of fractal parameters can thus be indicative of the temporal and spatial evolution of the fragmentation processes along a fault system in time and space. We analysed the seismological data with the software, fitting the same data with a linear regression, and obtained the fractal dimension and the related coefficient of determination (i.e., R-squared).

**Figure 5:**
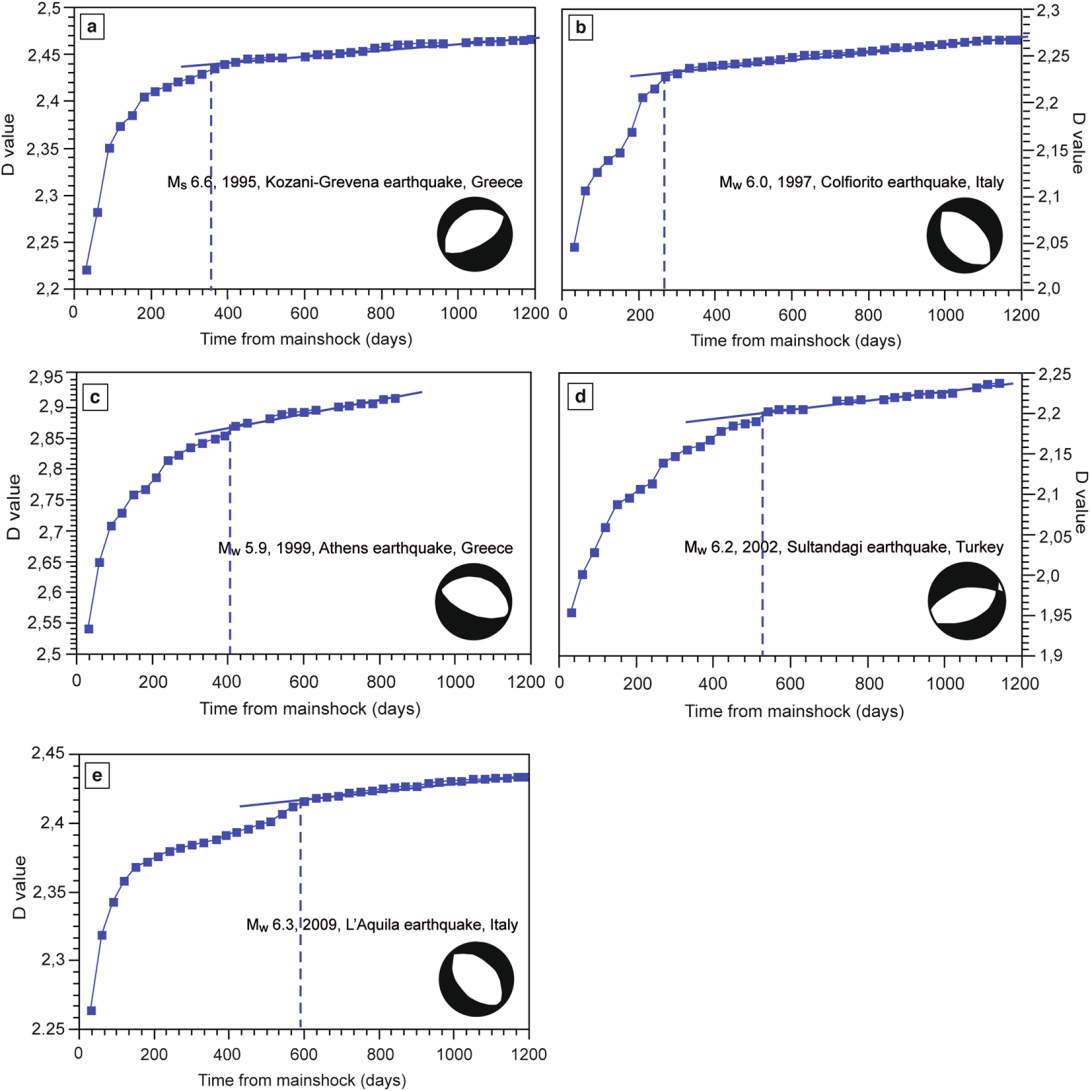
Aftershock sequences temporal evolution analysed by using the Mandelbrot method and following five mainshocks nucleated in extensional areas: (**a**) the M_s_ 6.6 Kozani-Grevena earthquake (1995, Greece), (**b**) the M_w_ 6.0 Colfiorito earthquake (1997, Central Italy), (**c**) the M_w_ 5.9 Athens earthquake (1999, Greece), (**d**) the M_w_ 6.2 Sultandagi earthquake (2002, Turkey), and (**e**) the M_w_ 6.3 L’Aquila earthquake (2009, Central Italy). The fractal dimension (D) values are shown versus the days from the mainshock nucleation.

**Figure 6:**
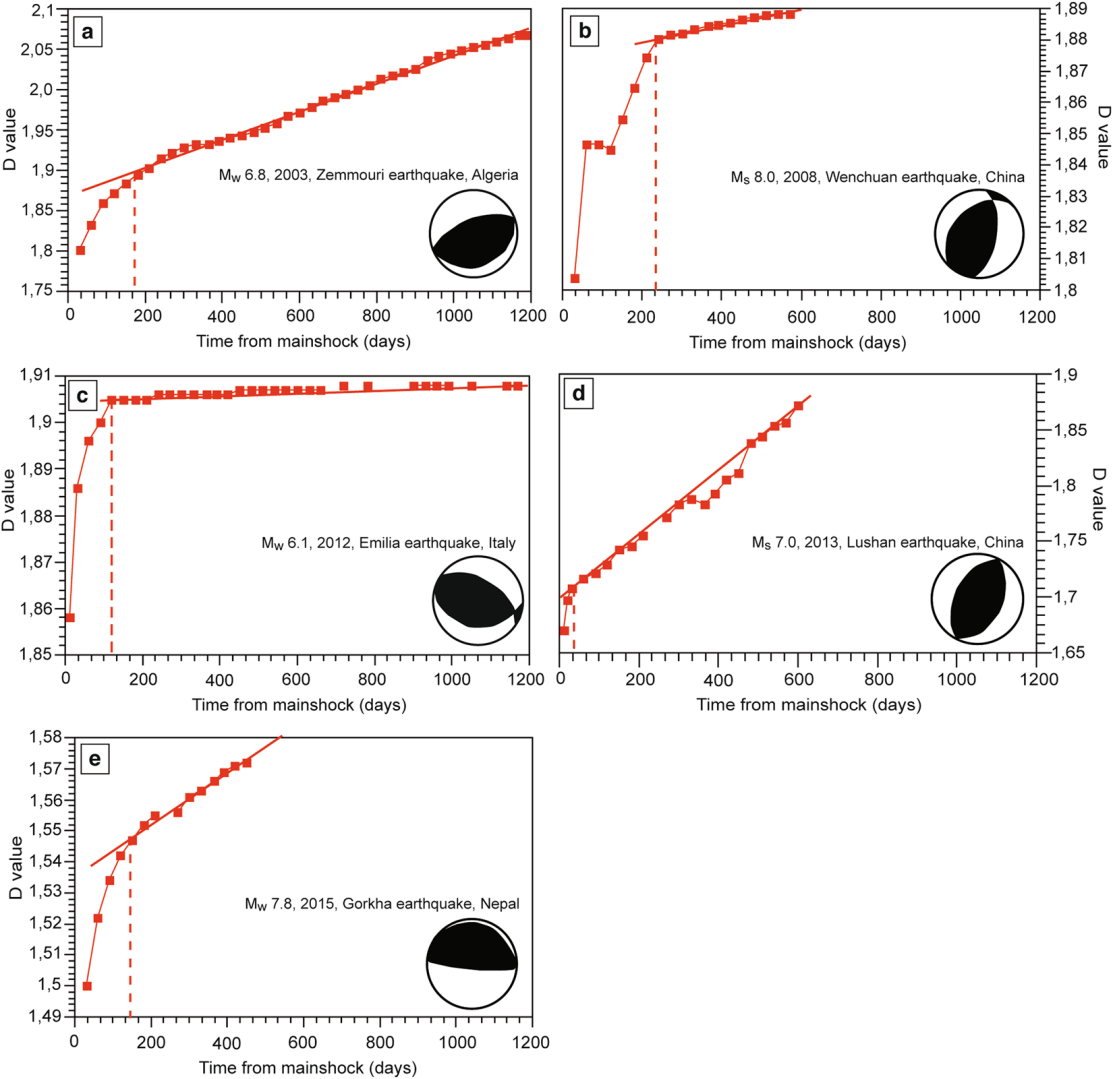
Aftershock sequences temporal evolution analysed by using the Mandelbrot method and following five mainshocks nucleated in compressional areas: (**a**) the M_w_ 6.8 Zemmouri earthquake (2003, Algeria), (**b**) the M_s_ 8.0 Wenchuan earthquake (2008, China), (**c**) the M_w_ 6.1 Emilia earthquake (2012, Northern Italy), (**d**) the M_s_ 7.0 Lushan earthquake (2013, China), and (**e**) the M_w_ 7.8 Gorkha earthquake (2015, Nepal). The fractal dimension (D) values are shown versus the days from the mainshock nucleation.

This method allows the representation of the magnitude-frequency distribution of earthquakes. We realized semi-logarithmic graphs for each seismic sequence, in which we compared the number of earthquakes occurred in certain magnitude ranges. The fitting straight line represents a simple linear regression according to the following equation, which also defines a fractal set:

4 $${N}_{i}=C{{r}_{i}}^{-D}$$

where ${N}_{i}$ is the number of objects with a characteristic linear dimension ${r}_{i}$, $C$ is a constant of proportionality, and $D$ is the fractal dimension^30^ (Figs 5 and 6). All these parameters directly derive from the regression lines automatically calculated by the used software. R-squared can range between 0 and 1. The higher the R-squared values, the higher the model accuracy. The high R-squared values in our studies (R^2^ > 0.95 in 90% of the cases and in one case R^2^ = 0.91) point to a good fit between the model accuracy and the data instability.

According to this topological analysis, the fractal dimension value represents the level of irregularity of the selected fractal set^29^ and is indicative of the fragmentation process occurred during the mainshock and the following aftershocks. If $D$ = 0, it represents the classical Euclidean dimension of a point; if $D$ = 1, the dimension of a line segment; if $D$ = 2, the dimension of a surface and, finally, if $D$ = 3, the Euclidean dimension of a volume^29,30^.

To check data accuracy, we calculated the coefficient of determination (R-squared) connected with Equation 4 for each seismic sequence (Figs S5,S6,S7 and S8). R-squared represents the variability between data instability and model accuracy. To establish the durations of aftershock sequences, we applied the Tangents method on the obtained fractal dimension trend.

## Results

Hereafter, we briefly describe the selected seismic events and report the results achieved for the duration of the seismic sequences (Table 1).

### Extensional tectonic setting

#### Kozani-Grevena earthquake (1995, Greece)

On May 13^rd^, 1995 a M_s_ 6.6 earthquake struck the Kozani-Grevena region (northwestern Greece). This area is located on the western margin of the Internal Hellenides fold-thrust belt. This fold-thrust belt underwent extension since at least the Pliocene, which generated NE-SW to ENE-WSW-trending normal faults^31,32^. The Kozani-Grevena mainshock occurred at 8.6 km depth along the ~8 km long Paleochori normal fault^33,34^.

According to the Tangents method, the sequence lasted about 300 days (Fig. 2a). The mainshock was followed by 989 aftershocks with M ≥ 2.5 (Fig. S9a). The strongest aftershocks occurred on July 17^th^, two months after the M_w_ 5.3 mainshock.

According to the Mandelbrot method the sequence lasted about 350 days, the fractal dimension (D) values vary from 2.22 to 2.47 (Fig. 5a) and theR^2^ is, on average, equal to 0.98 (Figs S6a and S8).

#### Colfiorito earthquake (1997, Central Italy)

On September 26^th^, 1997 a M_w_ 6.0 earthquake struck the Northern Apennines region, in Central Italy^35,36^. This area is located in the northern part of the Late Oligocene to present Apennines fold-thrust belt. Since middle Pliocene, the axial part of this fold-thrust belt underwent an extensional tectonics, which generated NW-SE-trending normal faults^37^. The Colfiorito mainshock occurred at 7.5 km depth along the ~12 km long Mt. Pennino-Mt. Prefoglio normal fault^38,39^, which belongs to this extensional setting.

According to the Tangents method the sequence lasted about 330 days (Fig. 2b). The mainshock was followed by 2119 aftershocks with M ≥ 2.5 (Fig. S9b). The strongest aftershocks occurred October 6^th^ and October 14^th^ with M_w_ 5.4 and M_w_ 5.6, respectively. Furthermore, six months later, on April 3^rd^, 1998 another M 5.1 earthquake nucleated.

According to the Mandelbrot method the sequence lasted about 330 days, D varies from 2.05 to 2.27 (Fig. 5b) and theR^2^ is, on average, equal to 0.96 (Figs S6b and S8).

#### Athens earthquake (1999, Greece)

On September 7^th^, 1999, a M_w_ 5.9 earthquake struck the city of Athens, in Greece. This area lies in between two Quaternary rift systems in central Greece: the Gulf of Corinth and the Gulf of Evia rifts^40^. E-W-trending and SW-dipping normal faults dominate the neotectonic structure of this region^41^. The Athens mainshock occurred at 9.5 km depth along the ~15 km long Parnitha normal fault^16,42,43^. The aftershocks activity was located at the eastern part of the activated normal fault^16^.

According to the Tangents method, the sequence lasted about 300 days (Fig. 2c). The mainshock was followed by 281 aftershocks with M ≥ 2.5 (Fig. S9c). The strongest M_w_ 4.8 aftershock occurred in the same day of the mainshock.

According to the Mandelbrot method, the sequence lasted about 400 days, D varies from 2.55 to 2.93 (Fig. 5c) and theR^2^ is, on average, equal to 0.99 (Figs S6c and S8).

#### Sultandagi earthquake (2002, Turkey)

On February 2^nd^, 2002 a M_w_ 6.2 earthquake struck the Sultandagi-Çay region of southwest Turkey^17^. This area is located in a complex geodynamic context, dominated by a series of graben and horst structures bounded by active oblique slip normal faults^17^. The main tectonic lineament of this region is represented by the undulated ~100 km long Sultandagi Fault, which trends primarily northwest^43^. The Sultandagi mainshock occurred at 7 km depth^17^ along this fault^45^.

According to the Tangents method, the sequence lasted about 500 days (Fig. 2d). The mainshock was followed by 922 aftershocks with M ≥ 2.5 (Fig. S9d). The strongest aftershocks occurred two hours after the mainshock with M_w_ 5.6.

According to the Mandelbrot method the sequence lasted about 540 days, D varies from 1.95 to 2.25 (Fig. 5d) and theR^2^ is, on average, equal to 0.9 (Figs S6d and S8).

#### L’Aquila earthquake (2009, Central Italy)

On April 6th, 2009 a M_w_ 6.3 earthquake struck the Central Apennines region, in Central Italy^46,47^. The L’Aquila mainshock occurred at 8 km depth along the ~15–18 km long Paganica normal fault^48^.

According to the Tangents method the seismic sequence lasted about 515 days (Fig. 2e). The mainshock was followed by 915 aftershocks with M ≥ 2.5 (Fig. S9e). The strongest aftershock occurred on April 7^th^ with M_w_ 5.4.

According to the Mandelbrot method, the sequence lasted about 560 days, D varies from 2.26 to 2.44 (Fig. 5e) and the R^2^ is, on average, equal to 0.98 (Figs S6e and S8).

### Case studies of contractional earthquakes

#### Zemmouri earthquake (2003, Algeria)

On May 21^st^, 2003 a M_w_ 6.8 earthquake struck the coastal region east of Algiers and the Tell Atlas, in Algeria^49^. The Zemmouri mainshock occurred at 9 km depth along a NE-SW trending thrust^50^, which is included in the thrust-and-fold Tell Atlas, in northern Algeria^51^.

According to the Tangents method the sequence lasted about 100 days (Fig. 3a). The mainshock was followed by 400 aftershocks with M ≥ 2.5 (Fig. S10a). The strongest aftershock occurred on May 27^th^, six days after the mainshock, with M_w_ 5.8.

According to the Mandelbrot method the sequence lasted about 165 days, D varies from 1.8 to 2.05 (Fig. 6a) and theR^2^ is, on average, equal to 0.97 (Figs S7a and S8).

#### Wenchuan earthquake (2008, China)

On May 12^th^, 2008 a M_s_ 8.0 earthquake struck the Sichuan Province, in central China. The main structural lineament of this area is located on the eastern edge of the Tibetan Plateau and is called the Longmenshan fault zone, which developed since Mesozoic time. This fault zone is about 500 km long, it dips to northwest and shows dextral transpressional kinematics^52^. The fault system is composed by four main faults: the back thrust, the central thrust, the front thrust and the blind thrust^52^. The Wenchuan mainshock occurred at 16 km depth along the central thrust^53,54^.

According to the Tangents method, the sequence lasted about 230 days (Fig. 3b). The mainshock was followed by 1859 aftershocks with M ≥ 2.5 (Fig. S10b). The strongest aftershock occurred on May 25^th^, thirteen days after the mainshock, with M_w_ 6.3.

According to the Mandelbrot method, the sequence lasted about 230 days, D varies from 1.8 to 1.89 (Fig. 6b) and theR^2^ is, on average, equal to 0.97 (Figs S7b and S8).

#### Emilia earthquake (2012, Northern Italy)

On May 20^th^, 2012 a M_w_ 6.1 earthquake struck the Po Plain, in Northern Italy. This area hosts the northernmost part of the Late Oligocene to present Apennines fold-thrust belt^55^. The Emilia mainshock occurred at 6.3 km depth along the 15 km long San Martino thrust^56^, which represents one of the active contractional structures buried under the Po Plain^57^.

According to the Tangents method, the sequence lasted about 110 days (Fig. 3c). The mainshock was followed by 782 aftershocks with M ≥ 2.5 (Fig. S10c). The strongest aftershock occurred on May 29^th^, nine days after the mainshock, with M_w_ 6.0, along the adjacent buried and seismogenic Mirandola thrust, at 10.2 km depth^56^.

According to the Mandelbrot method the sequence lasted about 120 days, D varies from 1.86 to 1.9 (Fig. 6c) and theR^2^ is, on average, equal to 0.97 (Figs S7c and S8).

#### Lushan earthquake (2013, China)

On April 20^th^, 2013 a M_s_ 7.0 earthquake struck again the Sichuan Province, in central China. The Lushan mainshock occurred five years after the Wenchuan earthquake along the same fault, at a depth of 15 km^22,52^.

According to the Tangents method the sequence was very short and lasted about 35 days (Fig. 3d). The mainshock was followed by 122 aftershocks with M ≥ 2.5 (Fig. S10d). The strongest aftershock occurred few minutes after the mainshock with M_w_ 5.4.

According to the Mandelbrot method the sequence lasted about 35 days, D varies from 1.67 to 1.87 (Fig. 6d) and theR^2^ is, on average, equal to 0.97 (Figs S7d and S8). We point out that, unlike the other case studies that show a typically curved trend, Fig. 5d shows a linear trend of the temporal evolution of the fractal dimension. This anomalous behaviour is related to the seismic catalogue incompleteness. In particular, the catalogue doesn’t include the seismic events with M < 3.0 and this could well justify the anomalous trend of the fractal dimension temporal evolution.

#### Gorkha earthquake (2015, Nepal)

On April 25^th^, 2015 a M_w_ 7.8 earthquake struck Nepal^23^. This region is inserted in the geodynamic context of the India-Eurasia collision. The Gorkha mainshock occurred at 15 km depth along the Main Himalayan Thrust^58^, a shallow dipping megathrust that accommodates most of the India–Eurasia convergence.

The aftershocks sequence propagated eastwards from the mainshock^59^ and, according to the Tangents method, lasted about 130 days (Fig. 3e). The mainshock was followed by 786 aftershocks with M ≥ 2.5 (Fig. S10e). The strongest aftershock occurred about thirty minutes after the mainshock with M_w_ 7.1.

According to the Mandelbrot method the sequence lasted about 130 days, D varies from 1.5 to 1.57 (Fig. 6e) and theR^2^ is, on average, equal to 0.96 (Figs S7e and S8).

## Discussion

The Tangents and Mandelbrot methods provide comparable durations for each seismic sequence (Table 1). The analysis of these sequences highlights the relationships between the aftershocks decay and the tectonic settings in which the seismic sequences occurred:

1) According to the Tangents method, the average duration of aftershock sequences within extensional and contractional tectonic settings are about 390 days and 120 days, respectively; extensional sequences are thus 270 days longer than contractional ones (Figs 2–4). Aftershock sequences within extensional tectonic settings comprise more seismic events (1045 aftershocks on average) than sequences within contractional earthquakes (790 aftershocks on average). Surprisingly, a M_w_ 6.0 extensional earthquake (i.e., Colfiorito earthquake, 1997, Italy) is characterized by a longer aftershocks sequence duration than a M_s_ 8.0 contractional earthquake (i.e., Wenchuan earthquake, 2008, China).

2) According to the Mandelbrot method, the average duration of aftershock sequences within extensional tectonic settings is about 430 days, 295 days longer than that of aftershock sequences within contractional tectonic settings (about 135 days; Figs 5 and 6). Furthermore, extensional aftershock sequences comprise more seismic events (1056 aftershocks on average) than those within contractional earthquakes (795 aftershocks on average).

The fractal dimension (D) values calculated for extensional and contractional sequences are also different. D varies between ca. 2–3 and ca. 1–2 for extensional and compressional earthquakes, respectively. As the fractal dimension is indicative of the geometrical fragmentation process, with time extensional seismic sequences are thus spatially distributed within a volume, while contractional seismic sequences closer to a fault along a surface. The average coefficient of determination R-squared (or R^2^) is, greater than 0.95 for nine seismic sequences (i.e., the 1995 Kozani-Grevena, the 1997 Colfiorito, the 1999 Athens, the 2009 L’Aquila extensional sequences and the 2003 Zemmouri, the 2008 Wenchuan, the 2012 Emilia, the 2013 Lushan and the 2015 Gorkha contractional sequences) and equal to 0.90 for the 2002 Sultandagi extensional sequence. These high R^2^ values indicate that the collected seismological data and the derived results are robust.

**Figure 7:**
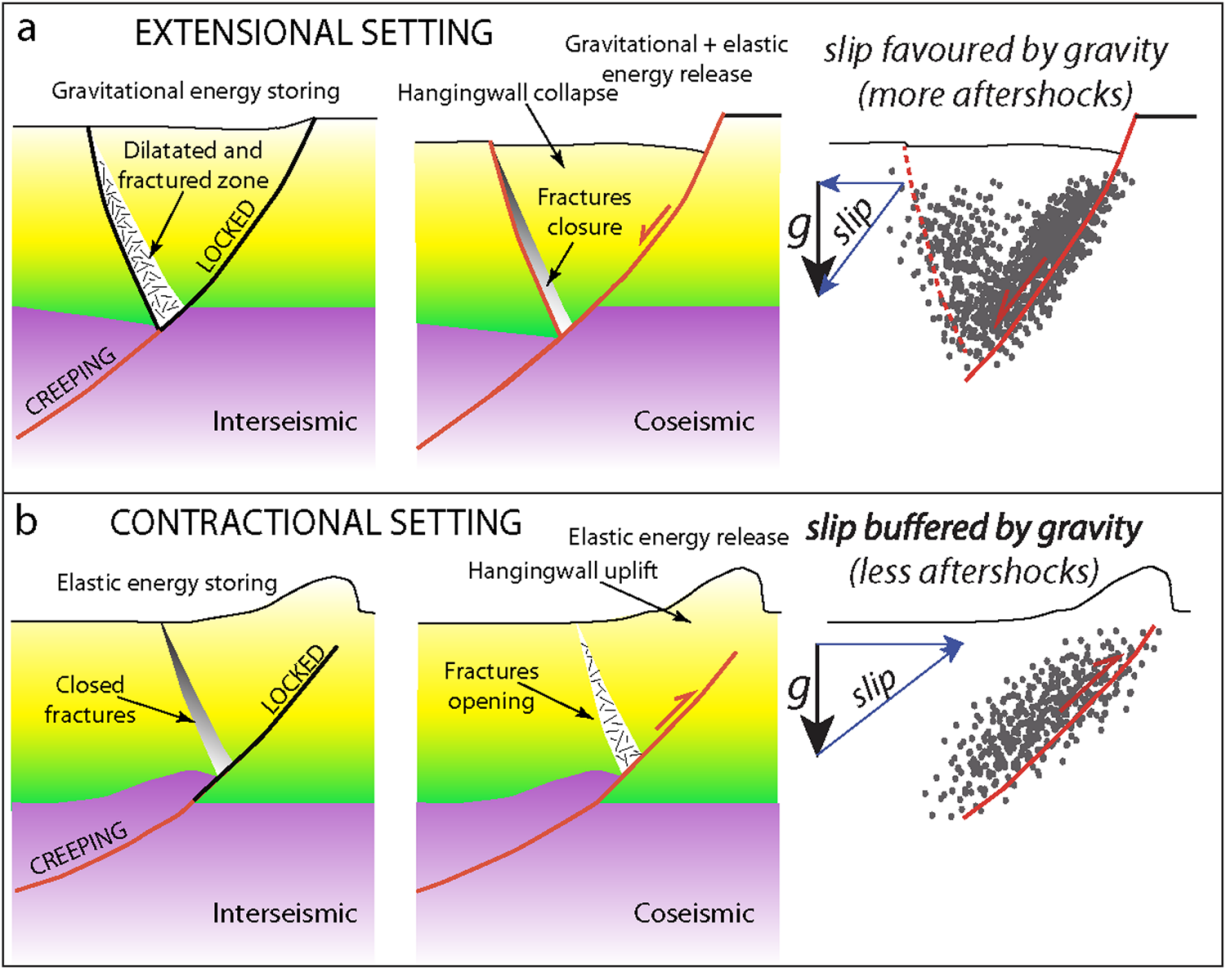
(**a**) Geological model of the seismic cycle (i.e., interseismic and coseismic periods) associated with a normal fault. (**b**) Geological model of the seismic cycle (i.e., interseismic and coseismic periods) associated with a thrust. In both models we assume a steady state strain rate in the ductile lower crust and a stick–slip motion in the brittle upper crust (modified from Doglioni *et al*.^62^). Extensional tectonic settings are characterized by longer aftershocks duration because the hangingwall moves in favour of gravity and the volume will collapse until a gravitational equilibrium is reached.

Our analyses strongly support the conclusion that, irrespective of the magnitude of the mainshocks, extensional seismic sequences are longer than compressional sequences. We propose that the type of energy released during the earthquakes, in turn related to the tectonic setting, controls the different duration of seismic sequences. The observed variability of the duration of aftershock sequences, however, could be conditioned by rock properties and fault characteristics. Doglioni *et al*.^9^ proposed that single extensional seismic sequences dissipate gravitational energy (Fig. 7a) stored during the interseismic phase, within a hangingwall volume confined by the main normal fault and an antithetic fractured dilated zone. When the stresses related to this gravitational energy exceed the strength of the dilated zone and of the main normal fault, the rock volume collapses slipping along the main fault, generating the earthquake^60^ (Fig. 7a). The downward hangingwall block movement is favoured by gravity^61^. This induces an increase of potential energy and facilitates fracturing processes both during the pre-seismic period and the coseismic phase. The potential energy is transformed into kinetic energy as indicated by the double-couple mechanism of the earthquakes generated by the shear on the fault planes. In this case, most of the involved forces are distributed within the fault hangingwall volume. After the onset of slip, the hangingwall preserves its inertia for a longer period since in this case external and internal (body) forces sum up downward. The aftershock sequences can be interpreted as related to the rock wedge settlement due to the closure of fractures and to the complete dissipation of gravitational energy within the dilated antithetic wedge^61,62^.

On the contrary, thrust-related earthquakes are characterized by dissipation of elastic energy^61,62^ (Fig. 7b), which is stored both within the rock volume above the thrust fault (i.e., the hangingwall block) and along the thrust fault itself during the pre-seismic period (Fig. 7b). When the elastic energy exceeds the fault resistance, the hangingwall block generates the earthquake by moving upward along the fault^57^. The elastic energy dissipation is buffered by the gravitational force. The downward directed gravitational force is opposite to the upward sense of motion of the fault hangingwall. As a consequence: (1) the work that the tectonic forces have to do on the block is greater and (2) after the onset of slip the hangingwall block preserves its inertia for a shorter period because most of the system energy is spent during rupture and slip along the main fault to overcome the gravitational force. For this reason (i.e., to overcome the inertial system), contractional earthquakes are of higher magnitude. Most of the energy stored during the interseismic phase is used to activate the main fault and stresses are concentrated at the interface between hangingwall and footwall blocks.

Since the amount of elastic energy necessary to move downward (i.e., in favour of the gravity force) the hangingwall block during extensional earthquakes is low, we suggest that aftershocks can be easily generated until gravitational equilibrium is reached again, producing longer extensional aftershock sequences. On the contrary, since the amount of elastic energy necessary to move upward (i.e., against the gravity force) the hangingwall block during compressional earthquakes is necessarily higher, the sequence of aftershocks can be interrupted earlier. Compressional aftershock sequences are thus shorter and comprise less earthquakes than in extensional aftershock sequences (Fig. 7). The physical model is based on both the versus and the different type of energy involved in extensional tectonic settings with respect to the contractional tectonic environments, as proposed in a previous study^61^.

## Conclusions

A comparative analysis of ten aftershock sequences occurred in extensional and contractional tectonic settings using two different methods (i.e., the Tangents method and the Mandelbrot method) allowed us to argue that, irrespective of the mainshock magnitude, aftershock sequences in extensional tectonic settings are longer comprised of more seismic events than those in contractional settings (Table 1).

We propose that these differences can be due to the different type of energy stored in extensional and contractional tectonic settings during the interseismic stage and dissipated during the earthquake sequences: (1) gravitational energy with a minor elastic component dominates in extensional sequences whereas (2) pure elastic energy is dissipated in contractional sequences. Therefore, for mainshocks of comparable magnitudes, extensional aftershock sequences last longer because the downward hangingwall movement is favoured by gravity and will continue until the gravitational equilibrium is reached. During contractional earthquakes, the upward hangingwall motion occurs instead against gravity and thrust-related aftershocks end earlier because the elastic energy dissipation is hindered by the gravitational force.

This comparative analysis of aftershock seismic sequences is useful in understanding the mid-term behaviour of an ongoing seismic sequence within different tectonic settings, providing useful inputs to improve seismic hazard assessment.

## Electronic supplementary material

Supplementary information
