# Supplementary material for: Longer aftershocks duration in extensional tectonic settings
Source: Sci Rep. 2017 Nov 27;7:16403. doi: 10.1038/s41598-017-14550-2 (PMC5703733; doi:10.1038/s41598-017-14550-2)
Supplement: Supplementary file 1 — Supplementary information [file 41598_2017_14550_MOESM1_ESM.pdf]

Longer aftershocks duration in extensional tectonic settings

E. Valerio[1], P. Tizzani[2,*], E. Carminati[1], C. Doglioni[1,3]

[1]Department of Earth Sciences, Sapienza University of Rome, Italy

[2]National Research Council (CNR), Istituto per il Rilevamento Elettromagnetico dell'Ambiente (IREA), Napoli, Italy

[3]Istituto Nazionale di Geofisica e Vulcanologia (INGV), Rome, Italy

*Corresponding author: tizzani.p@irea.cnr.it

Introduction

The Supplementary Information contains information about:

a) Aftershock sequences temporal evolution analysed by using the Omori law in case of five extensional earthquakes and five contractional earthquakes;

b) the strain release variations vs. time in case of five extensional earthquakes and five contractional earthquakes;

c) the R squared values versus time;

d) Magnitude distribution through time in case of five extensional earthquakes and five contractional earthquakes.

e) Areas evaluation: the Amatrice-Norcia extensional sequence vs. the Emilia contractional sequence

a) Aftershock sequences evolution according to the Omori law

We used ZMAP[1], an algorithm developed in MatLab environment, to describe the aftershocks decay of a seismic sequence according to the Omori-Utsu Law[2,3]:

$$n(t) = \frac{k}{(c+t)^p} \qquad \textit{(Eq. 1)}$$

where k and c are constant, t is the time and p indicates the rate decay. In particular, the p value is a key parameter representative of each earthquake and varies between 0.7 and 1.5. The variability in p-value may be related to the structural heterogeneity, stress and temperature in the crust[4]. However, it is not clear yet which of these factors is most significant in controlling the p-value. The K-value is dependent on the total number of events in the sequence and c on the rate of activity in the earliest part of the sequence. The constant c is a controversial quantity[4]; in fact, it is strongly influenced by the incompleteness of the catalogue in the early part of a sequence.

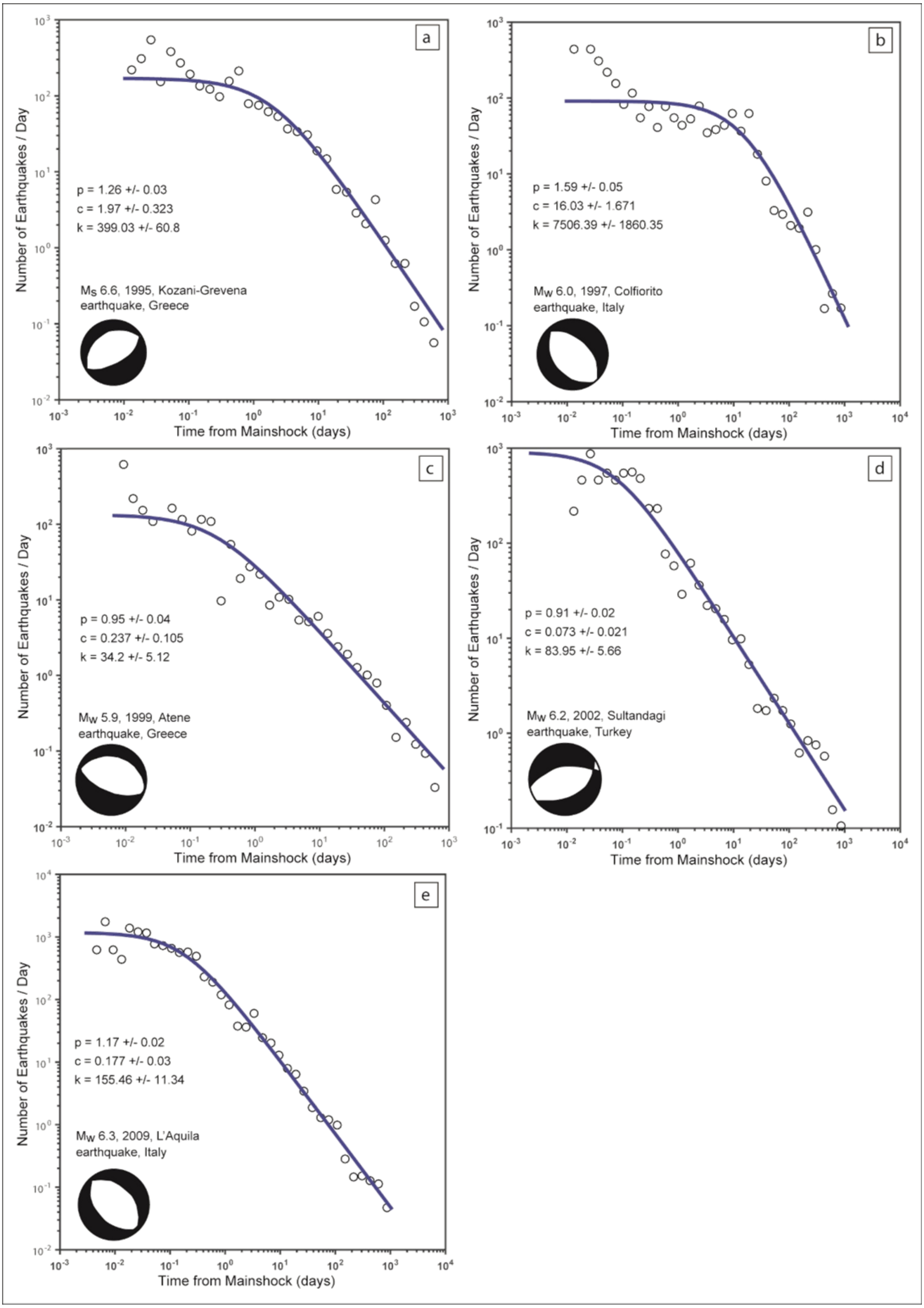

a
p = 1.26 +/- 0.03
c = 1.97 +/- 0.323
k = 399.03 +/- 60.8
Ms 6.6, 1995, Kozani-Grevena earthquake, Greece
b
p = 1.59 +/- 0.05
c = 16.03 +/- 1.671
k = 7506.39 +/- 1860.35
Mw 6.0, 1997, Colfiorito earthquake, Italy
c
p = 0.95 +/- 0.04
c = 0.237 +/- 0.105
k = 34.2 +/- 5.12
Mw 5.9, 1999, Atene earthquake, Greece
d
p = 0.91 +/- 0.02
c = 0.073 +/- 0.021
k = 83.95 +/- 5.66
Mw 6.2, 2002, Sultandagi earthquake, Turkey
e
p = 1.17 +/- 0.02
c = 0.177 +/- 0.03
k = 155.46 +/- 11.34
Mw 6.3, 2009, L'Aquila earthquake, Italy
Number of Earthquakes / Day
Time from Mainshock (days)

Supplementary Figure S1: Aftershock sequences temporal evolution analysed by using the Omori method in case of five extensional earthquakes case studies: a) the M_s 6.6 Kozani-Grevena earthquake (1995, Greece), b) the M_w 6.0 Colfiorito earthquake (1997, Central Italy), c) the M_w 5.9 Athens earthquake (1999, Greece), d) the M_w 6.2 Sultandagi earthquake (2002, Turkey), and e) the M_w 6.3 L'Aquila earthquake (2009, Central Italy). The fundamental parameter (p, c and k) values are also reported in the graphs. The days of the mainshock and after the mainshock are reported on the x-axis and the number of earthquakes per day is shown on the y-axis. The data are represented by circles and are fitted by a curve.

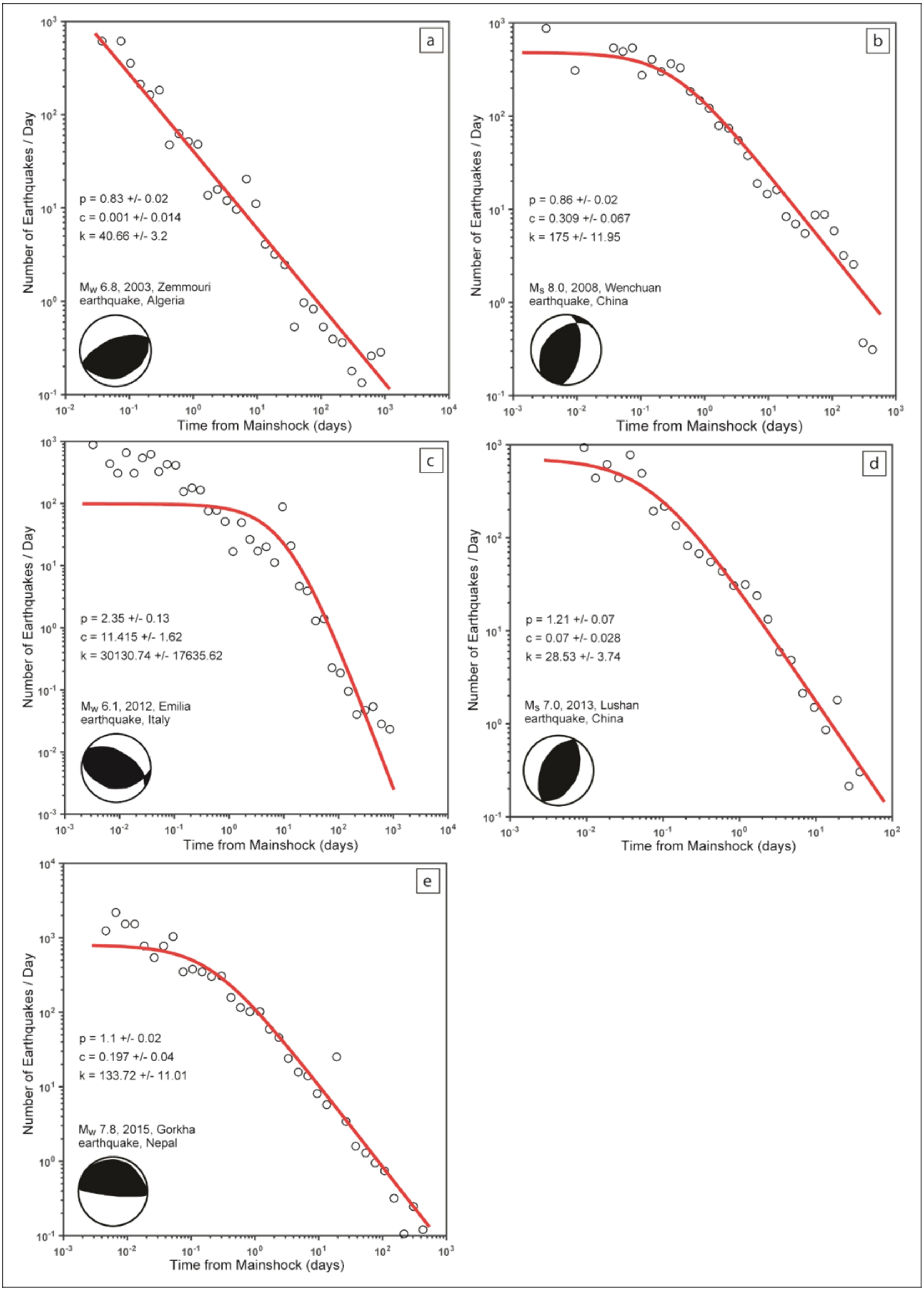
a
p = 0.83 +/- 0.02
c = 0.001 +/- 0.014
k = 40.66 +/- 3.2
Mw 6.8, 2003, Zemmouri earthquake, Algeria
b
p = 0.86 +/- 0.02
c = 0.309 +/- 0.067
k = 175 +/- 11.95
Ms 8.0, 2008, Wenchuan earthquake, China
c
p = 2.35 +/- 0.13
c = 11.415 +/- 1.62
k = 30130.74 +/- 17635.62
Mw 6.1, 2012, Emilia earthquake, Italy
d
p = 1.21 +/- 0.07
c = 0.07 +/- 0.028
k = 28.53 +/- 3.74
Ms 7.0, 2013, Lushan earthquake, China
e
p = 1.1 +/- 0.02
c = 0.197 +/- 0.04
k = 133.72 +/- 11.01
Mw 7.8, 2015, Gorkha earthquake, Nepal
Number of Earthquakes / Day
Time from Mainshock (days)

Supplementary Figure S2: Aftershock sequences temporal evolution analysed by using the Omori method in case of five compressional earthquakes case studies: a) the M_w 6.8 Zemmouri earthquake (2003, Algeria), b) the M_s 8.0 Wenchuan earthquake (2008, China), c) the M_w 6.1 Emilia earthquake (2012, Northern Italy), d) the M_s 7.0 Lushan earthquake (2013, China), and e) the M_w 7.8 Gorkha earthquake (2015, Nepal). The fundamental parameter (*p*, *c* and *k*) values are also reported in the graphs. The days of the mainshock and after the mainshock are reported on the x-axis and the number of earthquakes per day is shown on the *y*-axis. The data are represented by circles and are fitted by a curve.

	Extensional earthquakes		
	p	***c***	***k***
Kozani-Grevena earthquake	*1.26 ± 0.03*	*1.97±0.323*	*399.03±60.8*
Colfiorito earthquake	*1.59 ± 0.05*	*16.03 ± 1.671*	*7506.39 ± 1860.35*
Athens earthquake	*0.95 ± 0.04*	*0.237 ± 0.105*	*34.2 ± 5.12*
Sultandagi earthquake	*0.91 ± 0.02*	*0.073 ± 0.021*	*83.95 ± 5.66*
L'Aquila earthquake	*1.17 ± 0.02*	*0.177 ± 0.03*	*155.46 ± 11.34*
	Contractional earthquakes		
	p	***c***	***k***
Zemmouri earthquake	*0.83 ± 0.02*	*0.001 ± 0.014*	*40.66 ± 3.2*
Wenchuan earthquake	*0.86 ± 0.02*	*0.309 ± 0.067*	*175 ± 11.95*
Emilia earthquake	*2.35 ± 0.13*	*11.415 ± 1.62*	*30130.74 ± 17635.62*
Lushan earthquake	*1.21 ± 0.07*	*0.07 ± 0.028*	*28.53 ± 3.74*
Gorkha earthquake	*1.1 ± 0.02*	*0.197 ± 0.04*	*133.72 ± 11.01*

Supplementary Table S1: List of the parameters of the Omori Law (p, c and k) calculated for each seismic sequence.

b) Strain release variations vs. time

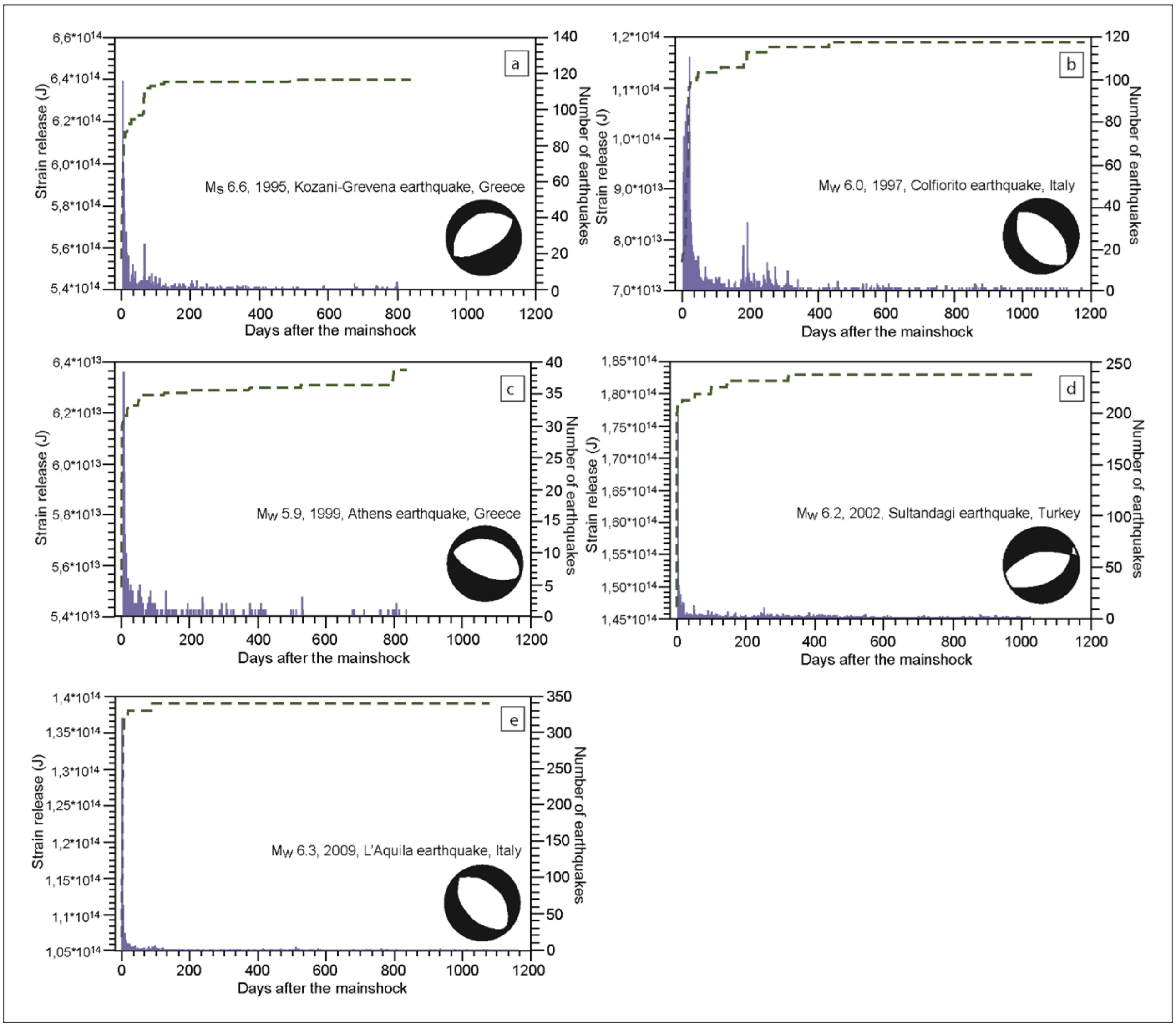

Supplementary Figure S3: The green dashed lines represent the strain release versus time and the number of occurred earthquakes in case of five extensional earthquakes: a) the M_s 6.6 Kozani-Grevena earthquake (1995, Greece), b) the M_w 6.0 Colfiorito earthquake (1997, Central Italy), c) the M_w 5.9 Athens earthquake (1999, Greece), d) the M_w 6.2 Sultandagi earthquake (2002, Turkey), and e) the M_w 6.3 L'Aquila earthquake (2009, Central Italy).

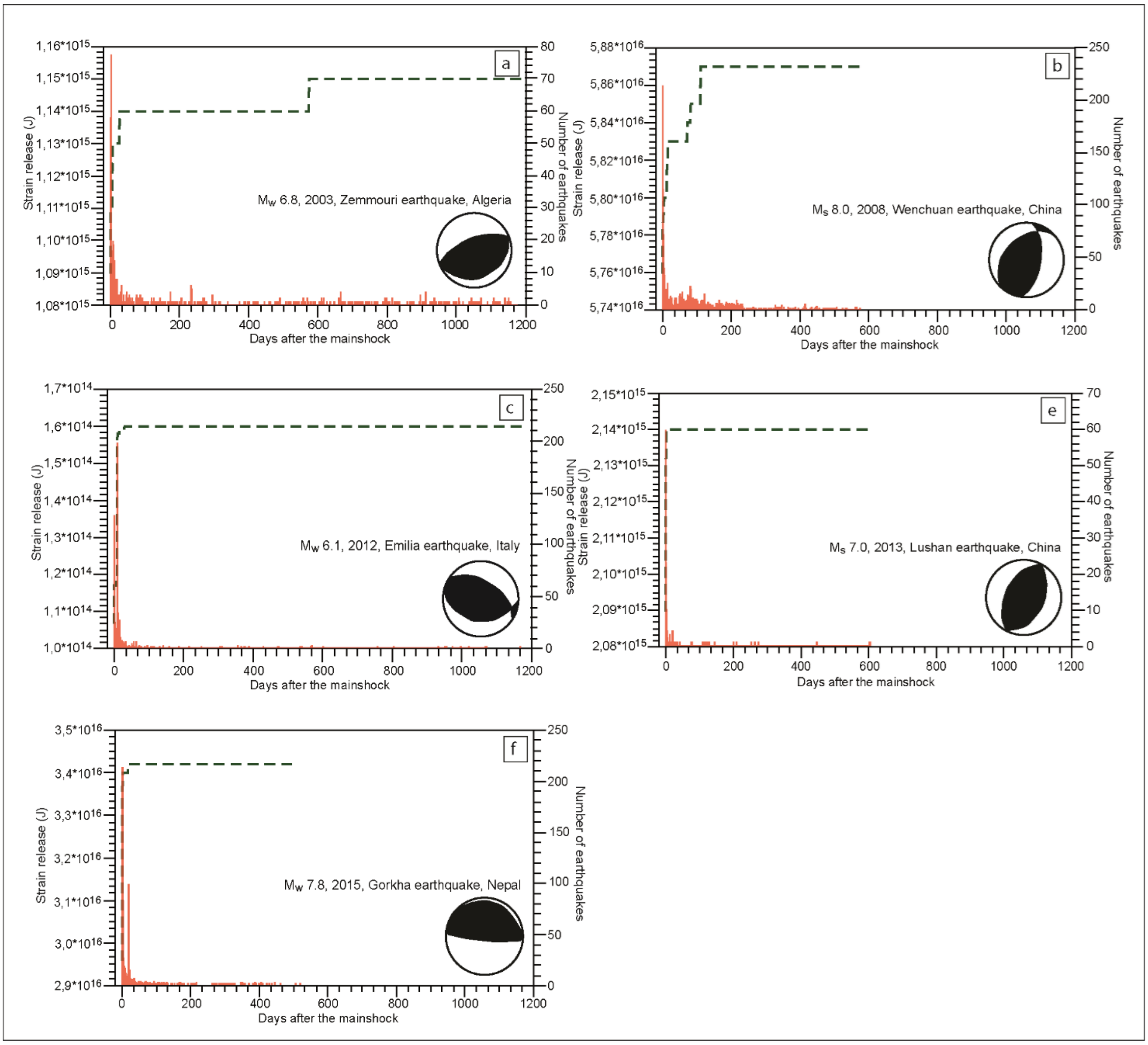

Supplementary Figure S4: The green dashed lines represent the strain release versus time and the number of occurred earthquakes in case of five compressional earthquakes: a) the M_w 6.8 Zemmouri earthquake (2003, Algeria), b) the M_s 8.0 Wenchuan earthquake (2008, China), c) the M_w 6.1 Emilia earthquake (2012, Northern Italy), d) the M_s 7.0 Lushan earthquake (2013, China), and e) the M_w 7.8 Gorkha earthquake (2015, Nepal).

c) R squared values

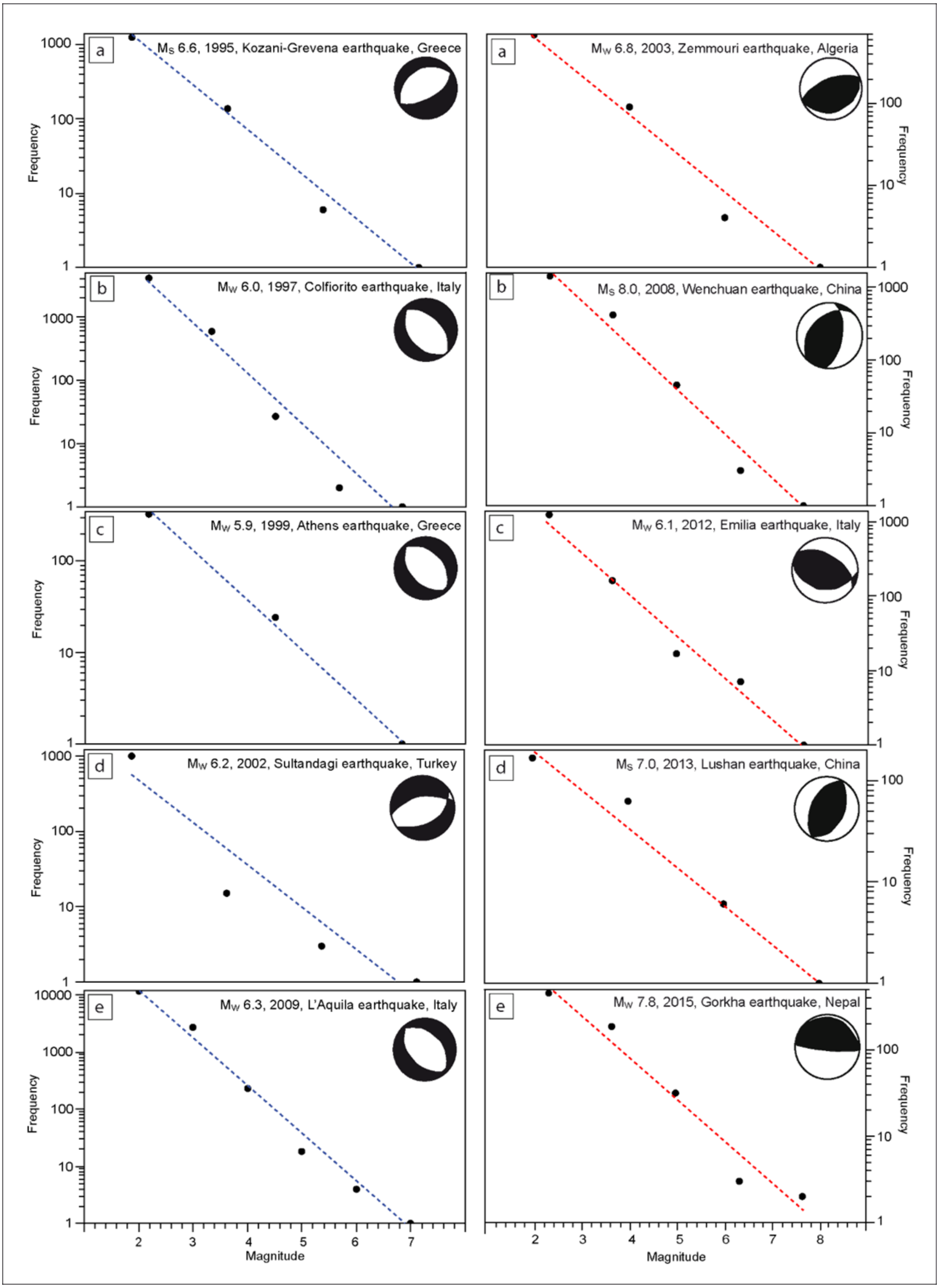

Supplementary Figure S5: Number of earthquakes versus magnitude calculated for each seismic sequence in correspondence of the maximum R squared value. Extensional earthquakes are reported on the left part of the graph and compressional earthquakes on the right part. In both cases the dashed lines follow the equation that define a fractal set and represent the linear regression.

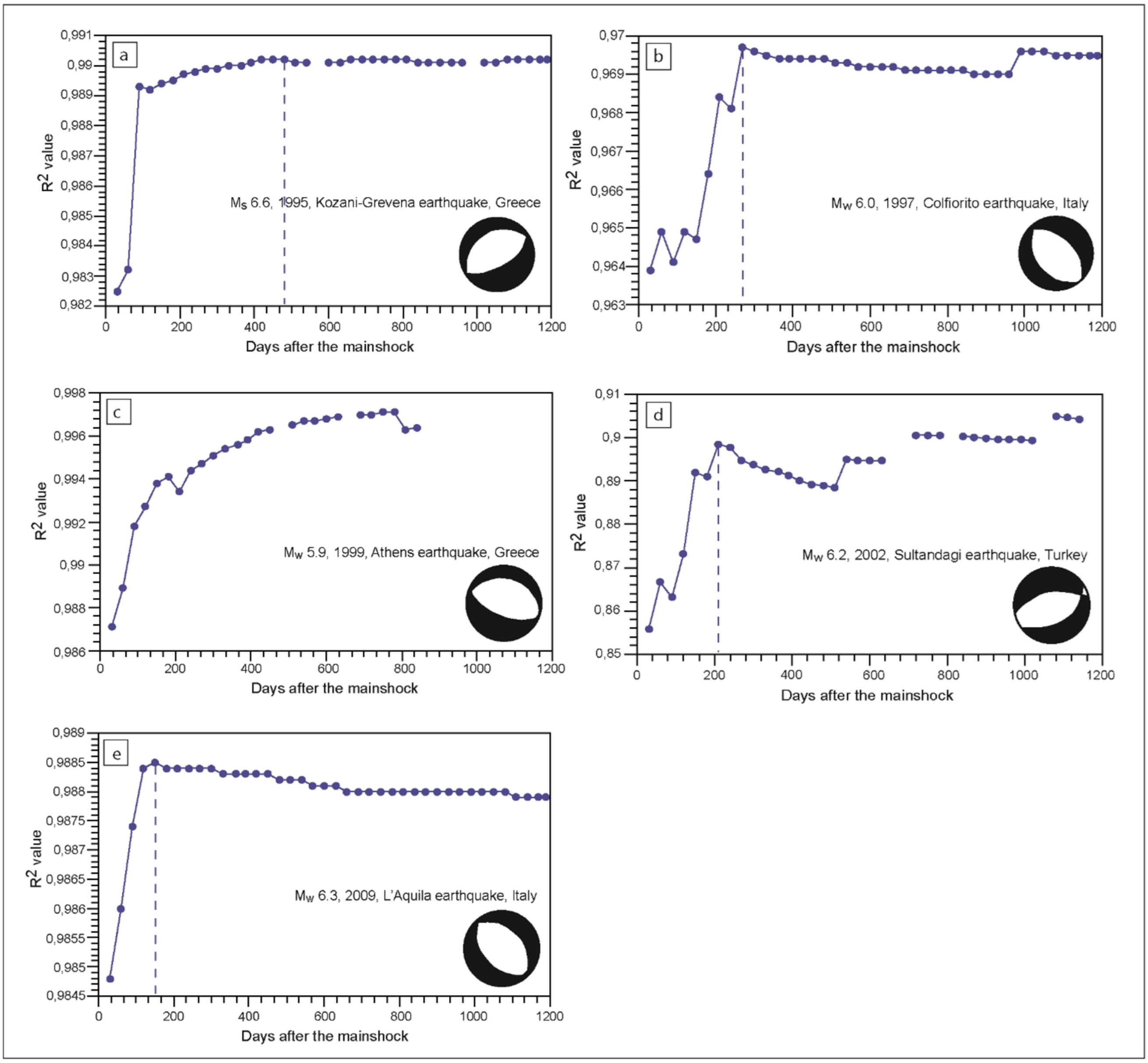

Supplementary Figure S6: R squared value temporal evolution in case of five extensional earthquakes: a) the M_s 6.6 Kozani-Grevena earthquake (1995, Greece), b) the M_w 6.0 Colfiorito earthquake (1997, Central Italy), c) the M_w 5.9 Athens earthquake (1999, Greece), d) the M_w 6.2 Sultandagi earthquake (2002, Turkey), and e) the M_w 6.3 L'Aquila earthquake (2009, Central Italy). The days of the mainshock and after the mainshock are reported on the x-axis and R squared (R^2) values are shown on the *y*-axis.

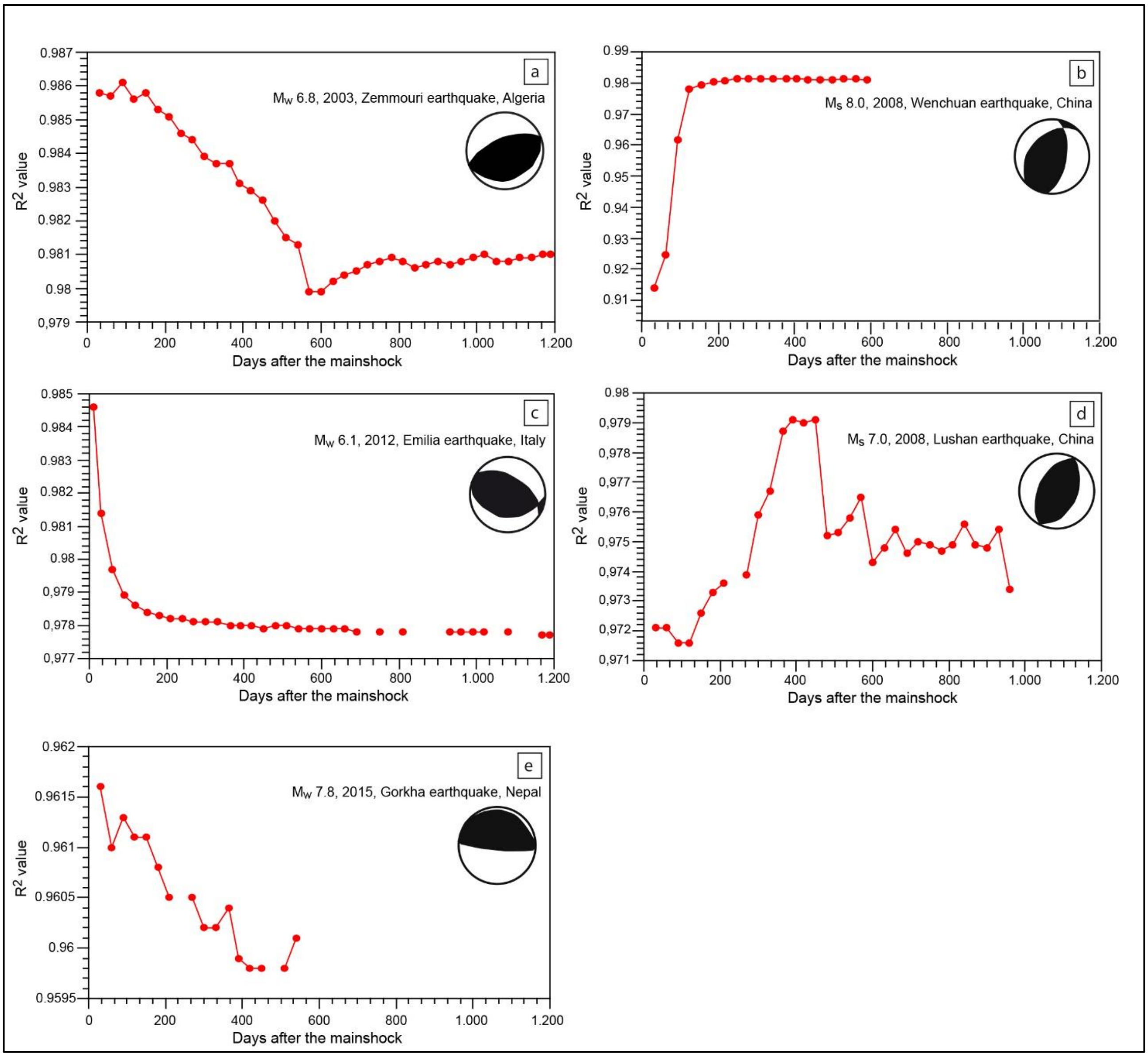

Supplementary Figure S7: R squared value temporal evolution in case of five compressional earthquakes: a) the M_w 6.8 Zemmouri earthquake (2003, Algeria), b) the M_s 8.0 Wenchuan earthquake (2008, China), c) the M_w 6.1 Emilia earthquake (2012, Northern Italy), d) the M_s 7.0 Lushan earthquake (2013, China), and e) the M_w 7.8 Gorkha earthquake (2015, Nepal). The days from the mainshock on are shown on the x-axis and the R squared (R^2) values are shown on the *y*-axis.

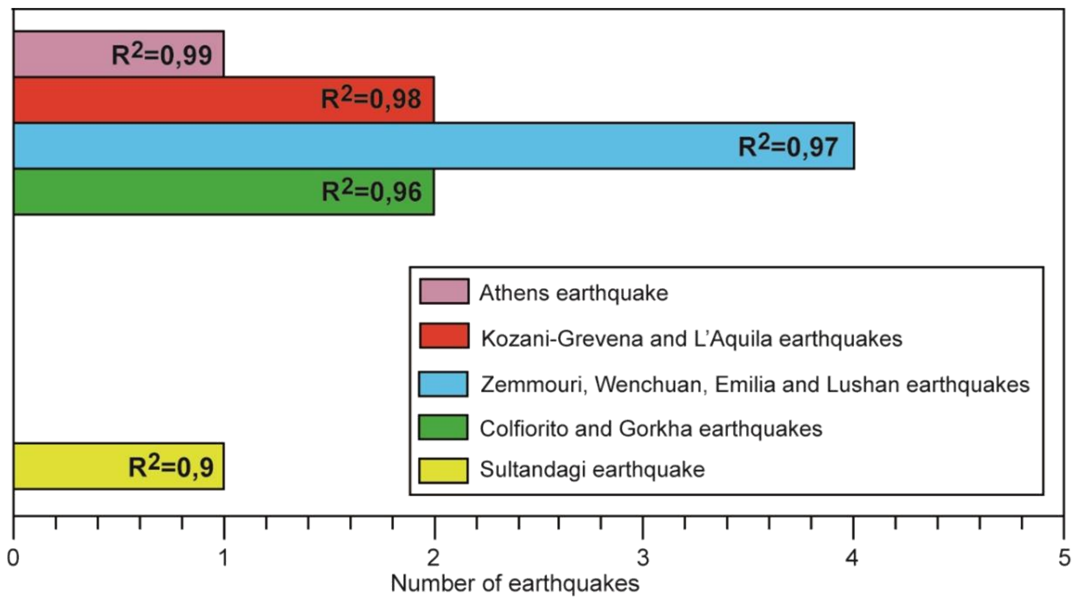

Supplementary Figure S8: Histogram of the R squared values calculated for each seismic sequence. The number of events is shown on the *x*-axis versus the R squared values reported on the *y*-axis. All the seismic sequences are characterized by $R^2 \geq 0.90$.

d) Magnitude distribution vs. time

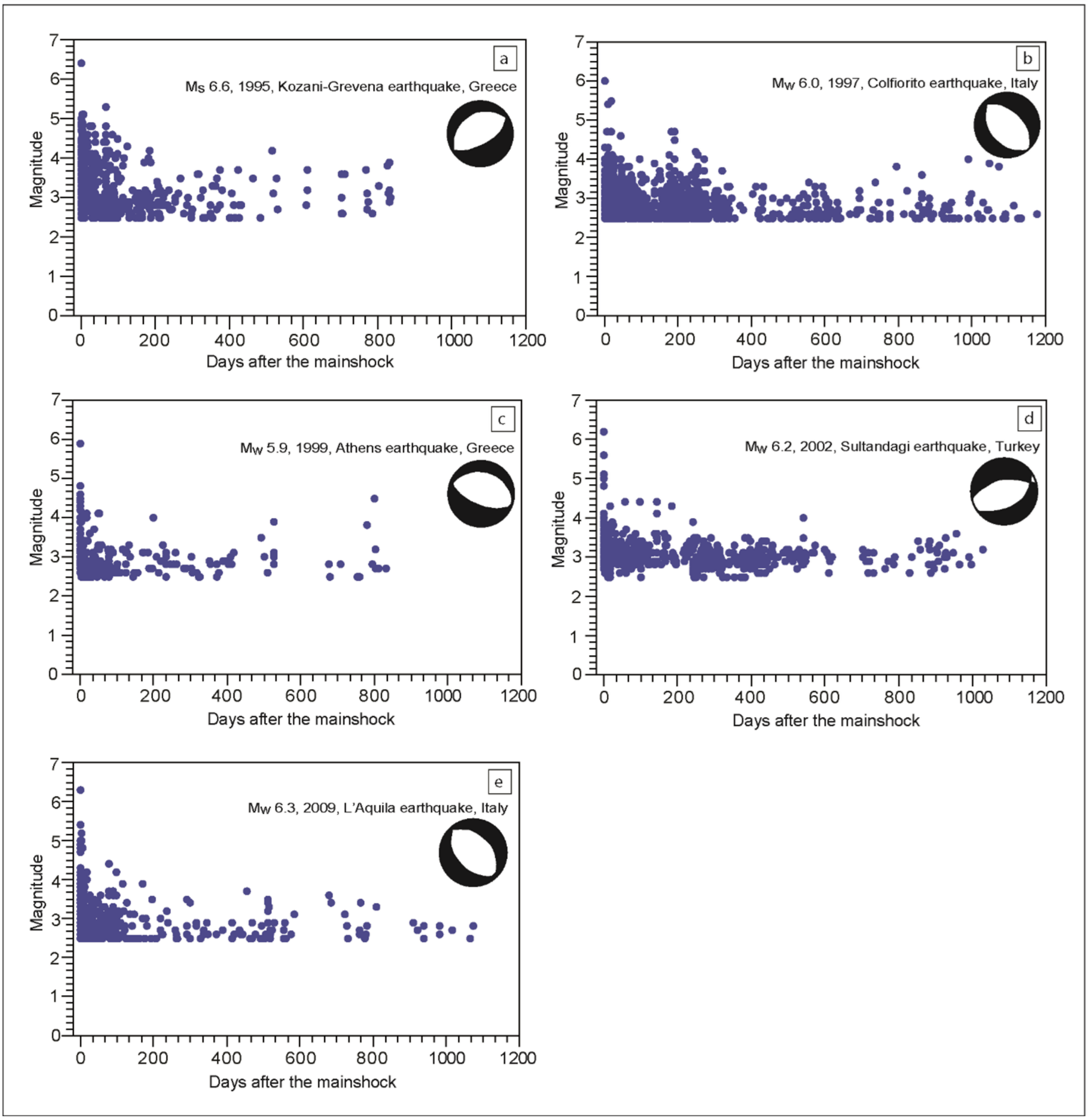

Supplementary Figure S9: Magnitude distribution versus time in case of five extensional earthquakes: a) the M_s 6.6 Kozani-Grevena earthquake (1995, Greece), b) the M_w 6.0 Colfiorito earthquake (1997, Italy), c) the M_w 5.9 Athens earthquake (1999, Greece), d) the M_w 6.2 Sultandagi earthquake (2002, Turkey), and e) the M_w 6.3 L'Aquila earthquake (2009, Italy). The days from the mainshock on are shown on the *x*-axis and magnitude values are shown on the *y*-axis.

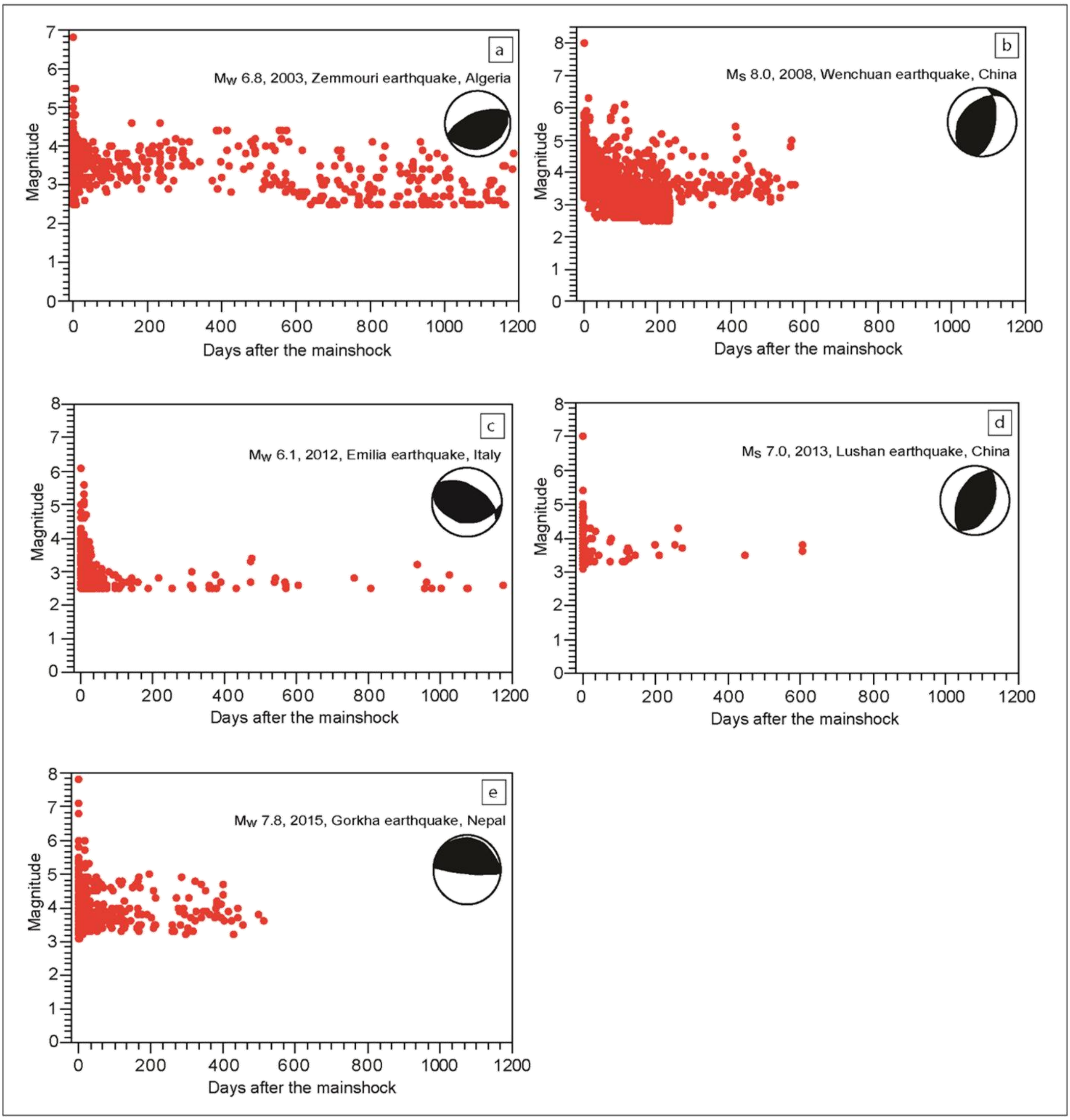

Supplementary Figure S10: Magnitude distribution versus time in case of five compressional earthquakes: a) the M_w 6.8 Zemmouri earthquake (2003, Algeria), b) the M_s 8.0 Wenchuan earthquake (2008, China), c) the M_w 6.1 Emilia earthquake (2012, Italy), d) the M_s 7.0 Lushan earthquake (2013, China), and e) the M_w 7.8 Gorkha earthquake (2015, Nepal). The days from the mainshock on are shown on the *x*-axis and magnitude values are shown on the *y*-axis.

e) Areas evaluation: the Amatrice-Norcia extensional sequence vs. the Emilia contractional sequence

We considered two case-studies: the Amatrice-Norcia extensional seismic sequence and the Emilia contractional seismic sequence. Both seismic sequences are characterized by the nucleation of two strong earthquakes. In the first case, the M_w 6.0 Amatrice earthquake occurred on August 24th, 2016 and was followed by the M_w 6.5 Norcia event nucleated on October 30th. In the second case, the M_w 6.1 Emilia mainshock occurred on May 20th, 2012 and, after nine days (on May 29th, 2012), a M_w 6.0 earthquake nucleated along the consecutive thrust.

We calculated the areas involved in the earthquake nucleation processes and suggest that the detected ground deformation pattern represents the surficial signature of deformation that affects a broad rock volume during extensional earthquakes and mostly along a surface during the evolution of contractional earthquakes, as also indicated by the fractal dimension analysis (Figs. 5 and 6). For this reason, we considered two images: the first one is the unwrapped interferogram of the Amatrice and Norcia extensional earthquake (Fig. S11a; 2016-2017, Central Italy, M_w 6.0 and M_w 6.5) and the second one is the unwrapped interferogram of the Emilia contractional earthquake (Fig. S11b; 2012, Northern Italy, M_w 6.1). We estimated the area included in the isolines -2 and +2 in case of the Amatrice-Norcia extensional sequence and of the Emilia contractional sequence, respectively. We obtained an area equal to ca. 350 km^2 for the Amatrice-Norcia case and equal to ca. 290 km^2 for the Emilia sequence.

The lateral extensions of the ground deformation pattern along the direction normal to the detected fault system show values included between 8-12 km and 5-7 km for the Amatrice-Norcia and the Emilia seismic sequences, respectively. Therefore, we argue that the subsidence volume involved in extensional earthquake nucleation processes is greater than the uplift volume involved in contractional nucleation processes.

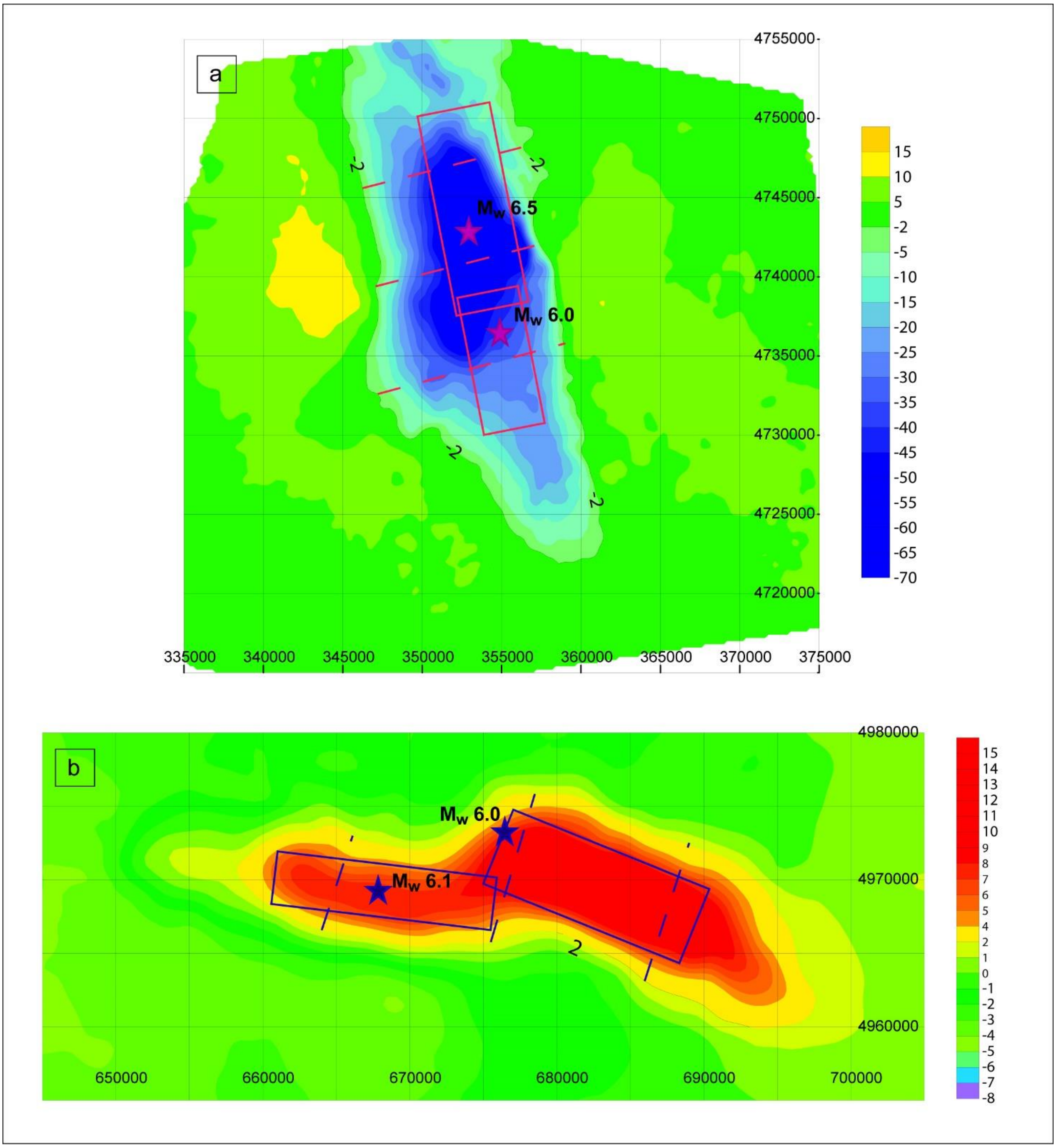

Supplementary Figure S11: a) Unwrapped interferogram of the Amatrice and Norcia mainshocks (2016, Central Italy, M_w 6.0 and 6.5, respectively). The magenta rectangles represent the fault structures along which the mainshocks (magenta stars) nucleate (e.g., Cheloni et al.[5]). b) Unwrapped interferogram of the Emilia contractional mainshocks (2012, Northern Italy, M_w 6.1 and 6.0, respectively). The blue rectangles represent the fault structures along which the mainshocks (blue stars) nucleate (e.g. Tizzani et al.[6]).

References

1. Wiemer, S. A software package to analyze seismicity: ZMAP. *Seism. Res. Lett.* **72**, 373-382 (2001).

2. Omori, F. On the after-shocks of earthquakes (Vol. 7). The University (1894).

3. Utsu, T. A statistical study of the occurrence of aftershocks. *Geophys. Magazine* **30,** 521–605 (1961).

4. Utsu, T., & Ogata, Y. The centenary of the Omori formula for a decay law of aftershock activity. *J. Phys. Earth* **43**, 1-33 (1995).

5. Cheloni, D. *et al.* Geodetic model of the 2016 Central Italy earthquake sequence inferred from InSAR and GPS data. Geophys. Res. Lett. (2017).

6. Tizzani, P. *et al.* New insights into the 2012 Emilia (Italy) seismic sequence through advanced numerical modeling of ground deformation InSAR measurements. *Geophys. Res. Lett.* **40**, 1971-1977 (2013).
